# Reference genes selection for *Calotropis procera* under different salt stress conditions

**DOI:** 10.1371/journal.pone.0215729

**Published:** 2019-04-18

**Authors:** Maria R. V. Coêlho, Rebeca Rivas, José Ribamar C. Ferreira-Neto, Valesca Pandolfi, João P. Bezerra-Neto, Ana Maria Benko-Iseppon, Mauro G. Santos

**Affiliations:** 1 Universidade Federal de Pernambuco, Departamento de Botânica, Laboratório de Fisiologia Vegetal, Recife, PE, Brazil; 2 Universidade Federal de Pernambuco, Departamento de Genética, Laboratório Genética e Biotecnologia Vegetal, Recife, PE, Brazil; Purdue University, UNITED STATES

## Abstract

*Calotropis procera* is a perennial Asian shrub with significant adaptation to adverse climate conditions and poor soils. Given its increased salt and drought stress tolerance, *C*. *procera* stands out as a powerful candidate to provide alternative genetic resources for biotechnological approaches. The qPCR (real-time quantitative polymerase chain reaction), widely recognized among the most accurate methods for quantifying gene expression, demands suitable reference genes (RGs) to avoid over- or underestimations of the relative expression and incorrect interpretation. This study aimed at evaluating the stability of ten RGs for normalization of gene expression of root and leaf of *C*. *procera* under different salt stress conditions and different collection times. The selected RGs were used on expression analysis of three target genes. Three independent experiments were carried out in greenhouse with young plants: i) Leaf_100_ = leaf samples collected 30 min, 2 h, 8 h and 45 days after NaCl-stress (100 mM NaCl); ii) Root_50_ and iii) Root_200_ = root samples collected 30 min, 2 h, 8 h and 1day after NaCl-stress (50 and 200 mM NaCl, respectively). Stability rank among the three algorithms used showed high agreement for the four most stable RGs. The four most stable RGs showed high congruence among all combination of collection time, for each software studied, with minor disagreements. *CYP23* was the best RG (rank of top four) for all experimental conditions (Leaf_100_, Root_50,_ and Root_200_). Using appropriated RGs, we validated the relative expression level of three differentially expressed target genes (*NAC78*, *CNBL4*, and *ND1*) in Leaf_100_ and Root_200_ samples. This study provides the first selection of stable reference genes for *C*. *procera* under salinity. Our results emphasize the need for caution when evaluating the stability RGs under different amplitude of variable factors.

## Introduction

*Calotropis procera* (Aiton) W. T. Aiton (Apocynaceae) is an evergreen shrub highly tolerant to drought and salt stresses with remarkable invasive ability in arid and semiarid regions [1]. Due to its pharmacognostic features, this shrub has been used in traditional medicine for the treatment of various diseases [1]. Ecophysiological studies have emphasized the superior physiology of *C*. *procera*, which show reduced stomatal conductance with high photosynthetic rate under water deficit [2,3]. These characteristics point this species as rich and attractive source of genes to be used in plant breeding programs for enhancing drought and salinity tolerance. In this sense, gene expression analysis can be used to evaluate the molecular mechanisms involved in plant response to different stresses. In the past years, advances in next-generation sequencing techniques have revolutionized transcriptomics and quickly established RNA-Seq as a robust methodology for gene expression analysis [4–7].

Efforts have focused on transcriptome and/or metabolomics of *C*. *procera* to study biosynthetic pathways of genes associated to the production of pharmacological compounds [8] and those involved in responses to heat, drought and salt stresses [9–11]. Because of its sensitivity, accuracy, reproducibility and rapid execution, qPCR has become a routine and robust approach for monitoring differential gene expression and validating data obtained by other methods, including RNA-Seq [12,13]. However, the accuracy of the qPCR results is largely influenced by RNA quality, cDNA preparation method and qPCR efficiency [14]. Such variables can cause quantitative and qualitative differences between the analyzed samples. Thus, a normalization step using endogenous controls [also called reference genes (RGs)] is essential [14–16]. RGs should ideally be constitutively expressed in the studied tissue or cell type and should not be affected by the treatments performed. Additionally, the uniform distribution of their transcripts across different treatments is required, functioning as a calibrator to compare different samples at the same quantitative level. The use of suitable RGs ensures the observed variation in target transcripts quantification is due to changes in expression, avoiding false positives or negatives in the process of gene expression analysis.

The most common RGs used in plants are those involved in fundamental cellular processes such as *actin* (*ACT*), *ubiquitin* (*UBQ*), *α-tubulin* (*TUA*), *β-tubulin* (*TUB*), *18S ribosomal RNA* (*18S rRNA*), *elongation factor 1-α* (*EF1α*), and *glyceraldehyde-3*-*phosphate dehydrogenase* (*GAPDH*) [17–19]. Nevertheless, there is evidence that the transcription level of commonly used RGs can vary considerably depending on the species, tissue type, developmental stage and, physiological and experimental conditions [16,20]. In this context, statistical algorithms such as geNorm [21], NormFinder [22], and BestKeeper [23] have been effectively employed to evaluate the best RGs for normalization of qPCR data.

Regarding *C*. *procera*, there are no reports to date on the selection of RGs previously submitted to a careful statistical analysis to determine their stability. In addition, considering the available reports, only one RG *GAPDH* [24] or *ACT* [8] has been used for qPCR assays in *C*. *procera*. This action reduces the statistical robustness the results. According to MIQE guidelines (Minimum Information for Publication of Quantitative real-time PCR Experiments) [25], the normalization step should be carried out against multiple RGs chosen from a variety of candidate RGs tested with the application of at least one algorithm.

This study verified the expression stability of 10 candidates RGs of *C*. *procera* in two tissue types (root and leaf) under different salt (NaCl) concentrations and different collect time combinations. Statistical algorithms, including geNorm, NormFinder, and BestKeeper, were used. In this sense, the present study provides the most stable and reliable RGs for each experimental condition. We also tested the selected RGs in the study of the three target genes expression in two tissue types (root and leaf), under different salt concentrations and different imposition of times.

## Materials and methods

### Plant material and salt stress assays

*Calotropis procera* seeds were collected on the seacoast of Pernambuco state, Brazil (7°50'32.9" S, 34°50'21.2" W, and 160 m away from the sea). Their surface was disinfected by immersion in 0.5% (v/v) sodium hypochlorite solution for 5 min. Seeds were germinated in Petri dishes with wet filter paper and kept in a growth chamber (at 25°C, 12 h photoperiod, and 70% relative humidity). After radicle emergence, seedlings were transferred to pots containing 7 kg of sandy soil and maintained in a greenhouse for three months. Plants were distributed in three independent experiments (S1 Table): i) Leaf_100_—young plants watered every day with NaCl (100 mM), during 45 days. At 30 min, 2 h, 8 h and 45 days of salt stress, youngest fully expanded leaves were collected; ii) Root_50_ and iii) Root200—young plants watered every day with NaCl (50 or 200 mM for Root_50_ and Root_200_, respectively). At 30 min, 2 h, 8 h and 1 day of salt stress, root tissue samples were collected. Control samples were watered daily with distilled water and collected for each salt stress time, respectively in each experiment. All samples were collected from three plant replicates. Samples were frozen in liquid nitrogen and stored at -80°C until RNA isolation.

### Total RNA isolation and cDNA synthesis

Total RNA was isolated from samples (leaf and root tissues) using the SV Total RNA Isolation System (Promega, Fitchburg WI, USA) by following the manufacturer’s instructions. RNA integrity was checked in 1.5% (w/v) agarose gel electrophoresis, stained with blue-green loading dye I (LGC Biotecnologia, SP, Brazil) and the quantity and quality of RNA samples were evaluated by fluorometry (Qubit, Oregon, USA). Reverse transcription reaction was carried out with 1 μg of total RNA, using the GoScript Reverse Transcription System Kit by (Promega, Fitchburg WI, USA) according to manufacturer’s instructions (Promega) and stored at -20°C.

### RNA-Seq libraries: Synthesis, sequencing, and analysis

We also performed transcriptome sequencing of *C*. *procera* leaves samples (NCBI Sequence Read Archive identification: PRJNA508417) exposed to NaCl (100 mM) at 30 min, 2 h, 8 h and 45 days after salt stress, including not stressed control samples (0 h and 45 days), according to the Leaf_100_ experiment description (S1 Table and S1 Fig). Each of the six RNA-Seq libraries was composed by a bulk combining equimolar RNA amounts of the three biological replicates were sequenced using Illumina paired-end sequencing technology on Illumina Hi-Seq TM 2500 platform (S1 Fig). After cleaning the raw reads and discarding low-quality reads, we ran Trinity [26] to assemble the clean reads into transcripts as described in Haas et al. [27].

Transcript quantification for RNA-Seq reads was performed with RSEM based on mapping the RNA-Seq reads of each experimental library (treatments 30 min, 2 h, 8 h compared to 0 h control and 45 days after stress imposition x 45 days control), against the assembled transcriptome [28] (S1 Fig). To estimate differential gene expression between our libraries, we used the edgeR tool [29], implemented in the Bioconductor package [30], requiring R software for statistical computing. The differentially expressed transcripts [log_2_Fold-Change (FC) > 2.0 or < - 2.0, and P-value < 0.05] were identified based on comparisons between experimental libraries and respective controls, using the normalized number of fragments mapping on each library. The ‘Fold-Change’ (FC) term afore-mentioned is a measure describing how much a quantity changes as compared with an initial (control) to a final value (treatment).

### Selection of target and candidate reference genes in the *C*. *procera* RNA-Seq libraries

Ten RGs were selected based on promising candidate genes according to previously published papers for other plant species [31–33], besides Log_2_FC between +1.0 and -1.0 and P-value > 0.05 for all the RNA-Seq expression contrasts (Table 1).

**Table 1:** Statistical parameters [Log_2_Fold-change (FC) and *P*-value] of the candidate reference genes (RGs) and target genes (TGs) selected from *Calotropis procera* leaf transcriptome (Illumina HiSeq 2500) under salt stress.

Category	Gene	30 min x Control (0 h)		2 h x Control (0 h)		8 h x Control (0 h)		45 d x Control (45 d)	
		FC	P-value	FC	P-value	FC	P-value	FC	P-value
RG	*MAPK2*	- 0.16	0.82	0.14	0.85	- 0.42	0.56	- 0.07	0.93
RG	*CYP23*	0.35	0.67	0.18	0.83	0.71	0.36	- 0.18	0.79
RG	*ACT104*	- 0.10	0.88	- 0.17	0.80	0.19	0.78	- 0.07	0.91
RG	*TBB4*	- 0.95	0.26	0.36	0.35	- 0.47	0.55	0.79	0.24
RG	*UBQ11*	0.44	0.51	1.00	0.11	0.77	0.24	- 0.34	0.60
RG	*ACT*	- 0.23	0.76	- 0.41	0.58	0.40	0.58	0.45	0.53
RG	*r40S*	0.11	0.87	0.03	0.97	1.00	0.50	- 0.12	0.86
RG	*PPR*	0.29	0.70	0.03	0.98	0.23	0.79	0.15	0.82
RG	*UBP25*	- 0.48	0.47	- 0.50	0.45	- 1.00	0.30	- 0.07	0.92
RG	*F-BOX*	- 0.27	0.73	0.28	0.68	0.13	0.87	- 0.69	0.30
TG	*ND1*	2.69 ^up^	0.02*	0.77	0.67	- 1.04	0.73	0.24	0.88
TG	*CNBL4*	0.63	1.00	1.22	0.44	2.75 ^up^	0.03^*^	- 1.14	0.35
TG	*NAC78*	2.74	0.12	2.61 ^up^	0.01^*^	3.61 ^up^	0.01^*^	0.33	0.71

* means statistical significance (*P* < 0.05),

^**up**^ Up-regulation of gene expression.

We selected, additionally, three target genes (TGs) related to salt stress response from the *C*. *procera* leaf transcriptome (RNA-Seq) to be used in qPCR gene expression analyses. TGs choice was based on two factors: (i) on their up-regulation (Log_2_FC > 2.0 and P-value < 0.05), at least one RNA-Seq expression contrast; and (ii) reported participation in the plant response to saline stress. The following TGs were scrutinized: *ND1* (*NADH dehydrogenase subunit 1* [34], *CNBL4* (*Calcineurin B-like protein 4* [35,36], and *NAC78* (*NAC domain-containing protein 78-like* [37,38] (Table 1).

Both RGs and TGs were submitted to the BLASTx (cut-off: e-value ≤ 3e -20) at NCBI [Non- redundant protein sequences (nr)] for annotation (Table 2).

**Table 2:** Primer pair of the candidate reference genes and target genes used in this study.

Gene	Description	Primer sequence (5’-3’)	Amplicon	T_a_ (°C)	*E* (%)		R^2^		BLASTx	
			(bp)		Leaf	Root	Leaf	Root	e-value	ID (%)
***Reference genes***										
***MAPK2***	*Mitogen-activated protein kinase kinase kinase 2*	F: AATGCTTCTGGGATTCTATGG R: CTTGATCCTATCTGTCGGAGA	94	62	101	100	0.998	0.987	3e-159	59
***CYP23***	*Peptidyl-prolyl cis-trans isomerase 23 isoform X2*	F: CATGTTCAATCCAGTCGAAGTC R: ATCATCCTTACCAGCAACCAGT	119	62	109	98	0.991	0.987	3e-50	95
***ACT104***	*Actin-104*	F: CACAATATGGCTGAGGGTGAG R: GAGCATCATCACCAGCAAATC	91	62	105	103	0.996	0.988	0.0	98
***TBB4***	*Tubulin beta-4 -chain*	F: CTTGCACCCTAACTCCACAAA R: CAACTTCCCAGAACTTTGATCC	99	62	107	102	0.998	0.999	0.0	95
***UBQ11***	*Polyubiquitin 11- like*	F: GGACCCTTGCTGACTATAATATCC R: CGTGAAGGAACTTAGACATGACC	88	62	101	105	0.994	0.998	7e-129	99
***ACT***	*Actin/actin-like conserved site-containing protein*	F: GAGGAGCACCCTATTCTTCTCA R: ACTGACTCCATCTCCAGAGTCC	186	62	102	108	0.995	0.991	2e-159	69
***r40S***	*40S ribossomal protein S3a*	F: ATACCAGTCCTTCTTGGCAAAC R: CGGGTGTATTTATGTGATGCAG	101	62	105	101	0.986	0.957	2e-88	95
***PPR***	*Putative pentatricopeptide repeat-containing protein*	F: ATTACCTTGCCTCATTCTGCTC R: AGAAGTCCTCCAGAGATGGTTG	148	62	100	111	0.999	0.999	0.0	73
***UBP25***	*Ubiquitin carboxyl-terminal hydrolase 25*	F: AATCACTTCTCTCACCGCTCTC R: ATCAGAGGGAGGGTGCTATTG	138	62	101	100	0.999	0.999	0.0	54
***F-BOX***	*F-box protein PP2-A12*	F: CCAGCAACACCACAGAAGAA R: AAGCAGGAAAGGGATTTGGT	143	62	107	108	0.994	0.998	2e-69	81
***Target genes***										
***ND1***	*NADH dehydrogenase subunit 1*	F: TTCAAGTATTGCTCCCGTTGGA R: CAGCGCGTAAACCACCTAAAAA	83	62	93	110	0.979	0.999	4e-33	97
***CNBL4***	*Calcineurin B-like protein 4*	F: CTTTTGCACGAGTCCGATCTTC R: AGCATCTCTGAACGTCTTATCCA	75	62	96	99	0.999	0.999	1e-45	75
***NAC78***	*NAC domain-containing protein 78-like*	F: TGGCGAAGGAAACGTTAGGTAT R: AATTCTCAAGTCTGTCGCCGAT	67	62	103	93	0.890	0.999	3e-20	39

T_a_, Anneling temperature; *E*, qPCR amplification efficiency; R^2^, regression coefficient; and ID: Identity.

### Primer design parameters

Transcript-specific primers were designed using the Primer3 web tool (http://bioinfo.ut.ee/primer3-0.4.0/) with the following parameter settings: length 18–22 bp, GC content of 45% - 55% (ideal content of 50%), annealing temperature (T_a_) of 58°C– 62°C (ideal of 60°C) and amplified products of 65–200 bp (Table 2).

### qPCR setup

The qPCR reactions were performed on PCR LineGene 9600 (Bioer, Hangzhou, China) using GoTaq qPCR Master Mix (Promega, Fitchburg WI, USA). Briefly, a 10 μL reaction mixture consisted of 5 μL *SYBR Green Super Mix* (Applied Biosystems, Foster City CA, USA), 2 μL of diluted cDNA (1/10), 0.3 μL for each primer (5 μM) and 2.4 μL ddH_2_O. Non-template controls were also included for each primer pair. Reactions were carried out under the following conditions: 95°C for 2 min, followed by 40 cycles of 95°C for 15 s and 62°C for 1 min. The melting curve was generated by varying the amplification temperature from 65–95°C. All qPCR reactions were carried out in triplicate (biological and technical) [25]. The amplification efficiency (*E*) was determined from a standard curve generated by serial dilutions of cDNA (1/10, 1/100, 1/1000, and 1/10000) for each primer, in triplicate, and calculated by using the equation: *E* = 10 ^(-1/slope of the standard curve)^ -1 [39]. Slopes in the range of -3.58 to -3.10 were considered acceptable for the qPCR assay [40]. These slope values correlated to amplification efficiencies between 90% (E = 1.9) and 110% (E = 2.1).

### Analysis of the reference genes expression stability

Three of the most notorious softwares available–geNorm v 3.5 [21], NormFinder v. 0.953 [22], and BestKeeper [23]–were used to evaluate the expression stability of ten candidate RGs: *ACT104*, *ACT*, *CYP23*, *FBOX*, *MAPK2*, *UBQ11*, *UBP25*, *PPR*, *r40S* and *TBB4* (Table 2). For geNorm and NormFinder, the raw Cq-values were transformed into relative quantities–Q = *E*^ΔCq^, where *E* represents the average efficiency for each gene, ΔCq is the difference between the lowest quantification cycle (Cq-value) of a sample of a particular gene and the Cq-value of each sample in a dataset [41].

In geNorm, the expression stability value (*M*) was calculated based on the average of the pairwise variation (*V*) for a candidate RG with all other genes tested, the default limit *M ≤* 1.5. Genes with the lowest *M*-value have the most stable expression [21]. The average *M* of all genes together is then calculated by stepwise exclusion of the least stable gene until the two most stable genes in the remaining set cannot be ranked any further. Besides, geNorm also allows estimating the optimal number of RGs that must be used for normalization process. Normalization factor (NF) is calculated based on the geometric mean of the expression of the two most stable RGs and then the NF_n+1_ with the next most stable gene. To determine the number of genes to be used for accurate normalization, the pairwise variation (*V*_n/n+1_) was determined out of two sequential NFs (NF_n_ and NF_n+1_) [21]. Vandesompele et al. proposed *V* ≤ 0.15 as a cut-off, below which the inclusion of an additional RG is not required [21].

NormFinder calculates the stability value using mathematical modeling algorithm to consider the intra- and inter-group variation of the candidate RGs. The lower stability value represents the highest stability. The fundamental principle is that a stable RG should have minimal variation across experimental groups and subgroups [22].

In BestKeeper, the raw Cq-values were used to calculate the Pearson correlation coefficient (*r*), which was obtained by the pairwise comparison between the BestKeeper index generated by the algorithm and the candidate RGs. Pearson correlation was determined as an indicator of expression stability, in which genes with higher *r*-value and P-value < 0.05 were more stable [23]. Samples with SD-value (standard deviation) > 1 were excluded from analysis [23]. Data from geNorm (*M*-values), NormFinder (stability values) and BestKeeper (*r*-value and SD) were used to generate rankings.

The expression stability of the candidate RGs was evaluated in all time combinations together: 30 min, 2 h, 8 h and 45 days, for Leaf_100_; 30 min, 2 h, 8 h and 1 day, for Root_50_ and Root_200._ Additionally, we also analyzed expression stability in a factorial time combination for each experiment, totaling 15 time combinations per experiment.

### Evaluation of target genes expression by qPCR

The expression pattern of three TGs (Table 1) was performed on Leaf_100_ (2 h, 8 h and 45 days) and Root_200_ (2 h, 8 h and 1 day) using the most stable candidate RGs suggested by the software applied. The Rest2009 software package (REST Standard mode) was used to calculate and analyze the relative expression of the TGs. Relative expression was calculated using the formula: *E* ^(ΔCq Target)^/ *E* ^(ΔCq RG)^, where *E* represents the average efficiency for each gene, ΔCq is the difference between mean Cq-value of a control sample and the mean Cq-value of treated sample. The REST bases its performance on pairwise comparisons (between RGs and TGs, control and treatment samples) using randomization and bootstrapping techniques (Pairwise Fixed Reallocation Randomization Test [42]. Hypothesis testing (*P* < 0.05) was used to determine whether the difference in expression between the control and treatment conditions was significant.

## Results

### Reference genes (RGs) and target genes (TGs) qPCR amplification

Ten candidates RGs were selected across *C*. *procera* RNA-Seq data, evaluated by qPCR and used to study the transcriptional modulation of three TGs. Products of these genes were associated with known functions involved in basal or vital cellular processes (Table 2). The specificity of PCR products was confirmed by the presence of a single amplicon with the expected size, with no amplicon visualized in non-template controls, as confirmed by 2% agarose gel electrophoresis (S2 Fig). The specificity of qPCR products was also confirmed by melting curves, each showing a single peak (S3 Fig).

**Fig 1:**
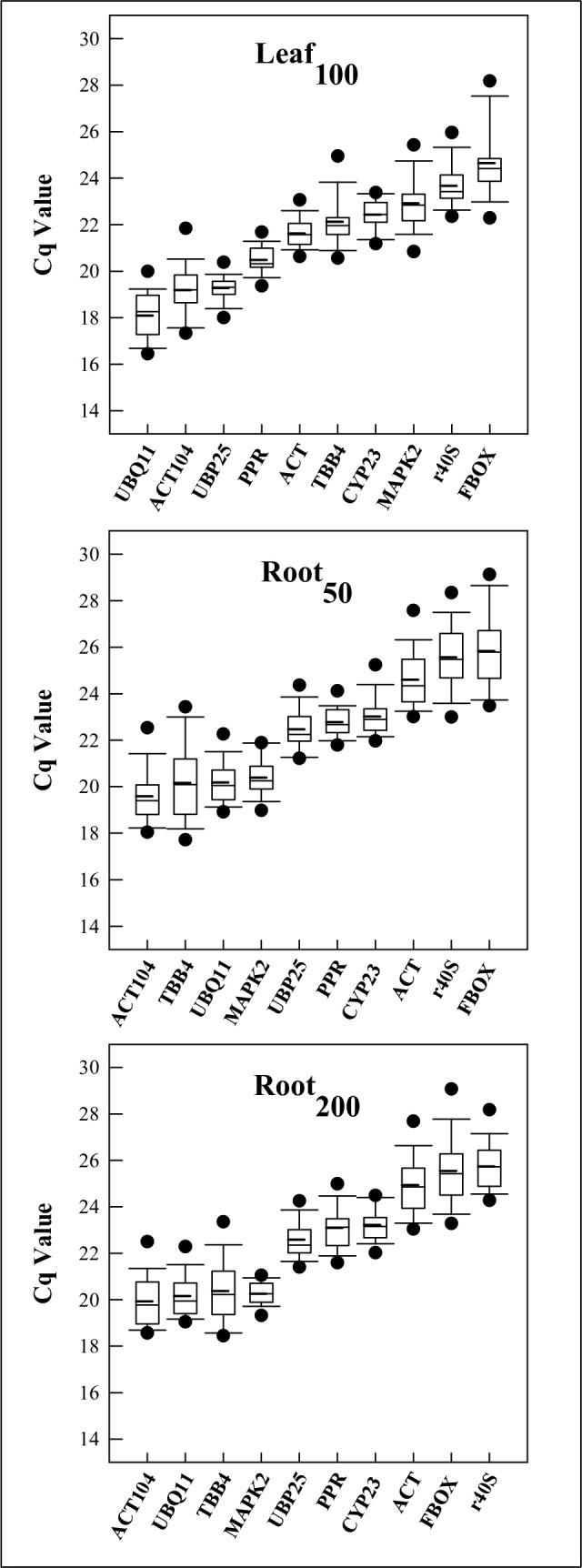
Quantification cycle (C_q_-value) of 10 candidate reference genes in leaf and root samples of *Calotropis procera* under different salt stress (A) Leaf_100_ (100 mM NaCl), (B) Root_50_ (50 mM NaCl) and (C) Root_200_ (200 mM NaCl). The Boxplot indicates the interquartile range. The horizontal dashed line represents the mean and the solid line the median. The upper and lower dashes represent the maximum and minimum values. Dots indicate the lowest and highest Cq value.

All RGs and TGs showed suitable amplification *E*-values, ranging from 93% (*ND1* and *NAC78*) to 109% (*CYP23*) (Table 2). The Cq-values provided by qPCR assay allowed us an overview of the gene expression levels (i.e., lower Cq-values correspond to higher expression levels and *vice-versa*). As shown in Fig 1, the mean Cq-values of ten RGs varied from 18.1 (*UBQ11* in Leaf_100_ samples) to 25.8 (*FBOX*, in Root_50_ samples) in all experiments. For Leaf_100_, the mean Cq-values ranged from 18.1–24.6 (*UBQ11* < *UBP25* < *ACT104* < *PPR* < *ACT* < *TBB4* < *CYP23* < *MAPK2* < *r40S* < *FBOX*, lower to higher Cq) (Fig 1A). The Cq-values of root samples was very similar in both experiments, with variation from ranging from 19.6–25.8 in Root_50_ (*ACT104* < *TBB4* < *UBQ11* < *MAPK2* < *UBP25* < *PPR* < *CYP23* < *ACT* < *r40S* < *FBOX*) (Fig 1B) and from 19.9–25.7 in Root_200_ (*ACT104*< *UBQ11* < *MAPK2* < *TBB4* < *UBP25* < *PPR* < *CYP23* < *ACT* < *FBOX* < *r40S*) (Fig 1C).

### Global analysis of expression stability

Considering all collection times together (global analysis) (30 min, 2 h, 8 h and 45 days for Leaf_100_; 30 min, 2 h, 8 h and 1 day for Root_50_ and Root_200_), the expression stability of each RG was analyzed to rank the most stable RGs for each experimental condition, using geNorm, NormFinder and BestKeeper algorithms (Tables 3 and S2).

**Table 3:** Ranking of the four most stable reference genes, according to geNorm, NormFinder and BestKeeper softwares, considering global in time combination of leaf and root samples of *Calotropis procera* under different salt stress: Leaf_100_ (100mM NaCl), Root_50_ (50mM NaCl) and Root_200_ (200mM NaCl).

	Assay	RANK				V-value	
		1^st^	2^nd^	3^rd^	4^th^	2/3	3/4
	**Leaf**_**100**_	*CYP23*	*ACT*	*PPR*	*r40S*	0.14	0.16
**geNorm**	**Root**_**50**_	*CYP23*	*UBP25*	*ACT104*	*ACT*	0.23	0.14
	**Root**_**200**_	*CYP23*	*UBP25*	*ACT104*	*ACT*	0.20	0.14
	**Leaf**_**100**_	*ACT*	*TBB4*	*PPR*	*r40S*		
**NormFinder**	**Root**_**50**_	*CYP23*	*UBP25*	*ACT104*	*UBQ11*		
	**Root**_**200**_	*UBP25*	*CYP23*	*ACT104*	*r40S*		
	**Leaf**_**100**_	TBB4*	ACT104*	r40S*	ACT*		
**BestKeeper**	**Root**_**50**_	ACT104*	CYP23*	UBP25*	ACT*		
	**Root**_**200**_	ACT104*	ACT*	UBP25*	CYP23*		

Leaf_100:_ leaf samples collected at 30 min, 2 h, 8 h and 45 days after 100 mM NaCl; Root_50_ and Root_200_: root samples collected at 30 min, 2 h, 8 h and 1 day after 50 and 200 mM NaCl, respectively. V-value, pairwise variation value. SD >1, genes excluded from the rank of BestKeeper; values followed by * variables do not depend linearly on each other are according to Pearson's correlation test (p < 0.05).

The geNorm algorithm showed *M*-value < 1.5 for all candidate RGs in all treatments (S2 Table). The four most stable RGs for NaCl-stressed leaves (Leaf_100_) were *CYP23*, *ACT*, *PPR*, and *r40S*, while *CYP23*, *UBP25*, *ACT104*, and *ACT* were most stable for NaCl-stressed roots (both Root_50_ and Root_200_) (Table 3). On the other hand, the less stable RGs were *UBP25* and *UBQ11* for Leaf_100_; *FBOX* and *TBB4* for Root_50_; *FBOX* and *TBB4* for Root_200_ were the less stable RGs (S2 Table).

According to the NormFinder algorithm, the four most stable RGs were *ACT*, *TBB4*, *PPR* and *r40S* in Leaf_100_; *CYP23*, *UBP25*, *ACT104* and *UBQ11* in Root_50_ and *UBP25*, *CYP23*, *ACT104* and *r40S* in Root_200_ (Table 3). The less stable RGs in Leaf_100_ were *UBQ11* and *UBP25*; for Root_50_ were *FBOX* and *TBB4*, and for Root_200_ were *FBOX* and *TBB4* (S2 Table).

For BestKeeper algorithm, the four most stable RGs were *TBB4*, *ACT104*, *r40S* and *ACT* in Leaf_100_; *ACT104*, *CYP23*, *UBP25* and *ACT* in Root_50_ and *ACT104*, *ACT*, *UBP25* and *CYP23* in Root_200_ (Table 3). The less stable RGs were *FBOX* and *UBP25* for Leaf_100_; *FBOX* and *r40S* for Root_50_; *FBOX* and *TBB4* for Root_200_ (S2 Table).

**Fig 2:**
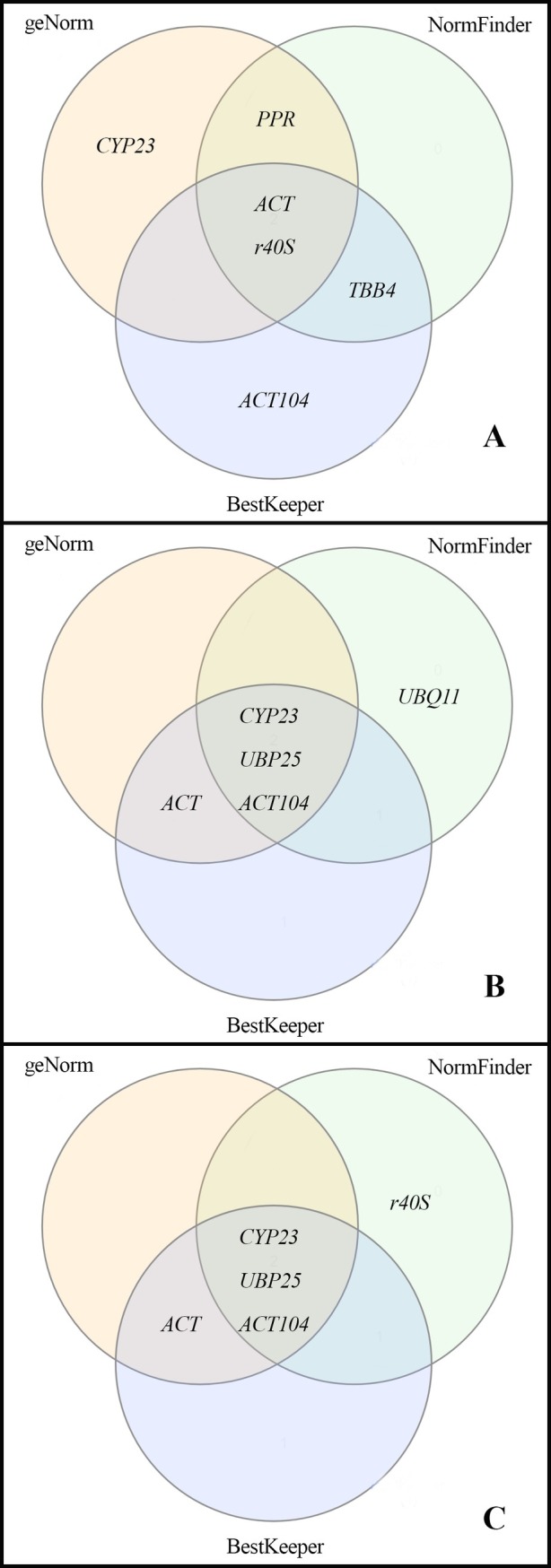
Comparison among geNorm, NormFinder and BestKeeper concerning to four top-ranked shared reference genes using all sampling times together (global analysis) of leaf and root samples of *Calotropis procera* under different salt stress: A) Leaf_100_ (100mM NaCl); B) Root_50_ (50mM NaCl); and C) Root_200_ (200mM NaCl).

Although each software has its own statistical method to provide a stability rank, there is a certain degree of congruence among their results. In the current study, the congruence among geNorm, NormFinder, and BestKeeper is presented, concerning the four top-ranked RGs using all collect times together (global analysis) (Fig 2). For Leaf_100_ samples, we observed 75%, 75% and 50% congruence between geNorm *vs*. NormFinder, NormFinder *vs*. BestKeeper and geNorm *vs*. BestKeeper, respectively (Fig 2A). In turn, we had congruence for root samples (Root_50_ and Root_200_) between geNorm *vs*. NormFinder, NormFinder *vs*. BestKeeper and geNorm *vs*. BestKeeper, corresponding to 75%, 75%, and 100%, respectively (Fig 2B and 2C).

The RGs choice to use in qPCR analysis was determined according to geNorm, which also provided high congruence between other softwares studied and presented the optimal number of RGs required for reliable normalization according to *V*-value ≤ 0.15. In this context, for Leaf_100_ (30min, 2 h, 8 h, and 45 d), geNorm determined two RGs (*CYP23* and *ACT*) as the best pair (Table 3). On the other hand, geNorm determined three RGs (*CYP23*, *UBP25*, and *ACT104*; see V_3/4_ ≤ 0.15; Table 3) as most suitable RGs for Root_50_ and Root_200_ (30 min, 2 h, 8 h, and 1 d).

### Analysis of the expression stability considering factorial time combination

**Fig 3:**
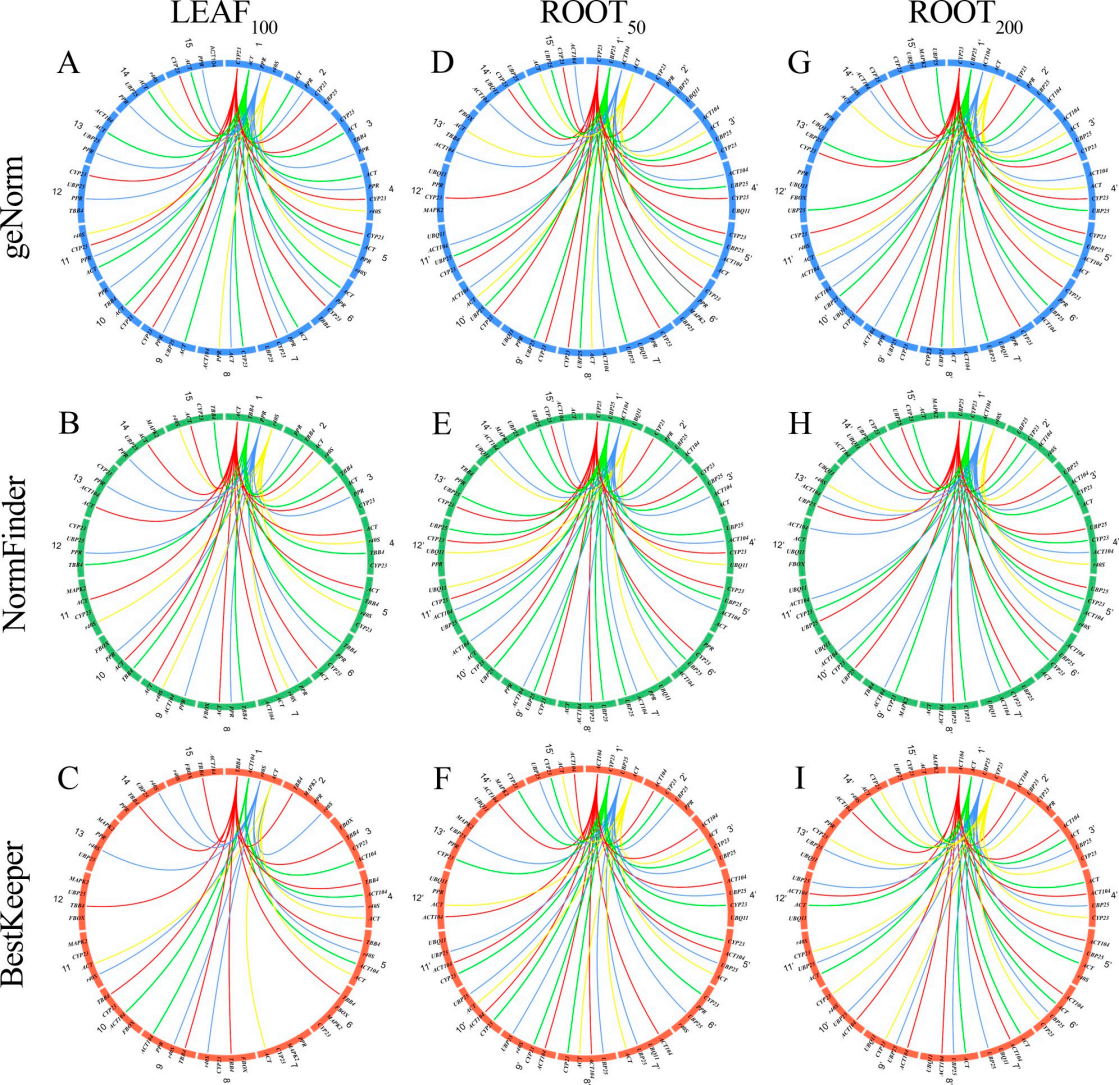
Comparison among 14 time combinations (2–15 for leaf samples and 2’-15’ for root samples) concerning the global time combination (1) of four top-ranked reference genes in geNorm (A, D, G), NormFinder (B, E, H) and BestKeeper (C, F, I) of leaf and root samples of *Calotropis procera* under different salt stress: Leaf_100_ (100 mM NaCl), Root_50_ (50 mM NaCl) and Root_200_ (200 mM NaCl). Numbers represent time combinations: Leaf_100_: 1 (30min-2h-8h-45d), 2 (30min-2h-8h) 3 (30min-2h-45d), 4 (30min-8h-45d), 5 (2h-8h-45d), 6 (30min-2h), 7 (30mim-8h), 8 (30min-45d), 9 (2h-8h), 10 (2h-45d), 11 (8h-45d), 12 (30min), 13 (2h), 14 (8h), 15 (45d). Root_50_ and Root_200_: 1’ (30min-2h-8h-1d), 2’ (30min-2h-8h) 3’ (30min-2h-1d), 4’ (30min-8h-1d), 5’ (2h-8h-1d), 6’ (30min-2h), 7’ (30mim-8h), 8’ (30min-1d), 9’ (2h-8h), 10’ (2h-1d), 11’ (8h-1d), 12’ (30min), 13’ (2h), 14’ (8h), 15’ (1d).

We also evaluated expression stability of the RGs per factorial combination from all collection times for each experiment, totaling 15-time combinations (S2 Table). Comparing all 15-time combinations in each algorithm revealed that the four most stable RGs are not strictly preserved for Leaf_100_, Root_50,_ and Root_200_ (Fig 3 and S2 Table). In this context, we averaged the congruence of all different collection times compared to global collection time (30 min, 2 h, 8 h and 45 days for Leaf_100_; 30 min, 2 h, 8 h and 1 day for Root_50_ and Root_200_). For Leaf_100_ samples, we observed on average 84%, 70% and 54% of congruence concerning global time combinations for geNorm, NormFinder and BestKeeper, respectively (Fig 3A–3C and S2 Table). On the other hand, concerning to global time combinations for Root_50_, average congruence for geNorm, NormFinder and BestKeeper was 71%, 77%, and 77%, respectively (Fig 3D–3F and S2 Table). Moreover, we observed average congruence of 73%, 73%, and 79% for geNorm, NormFinder, and BestKeeper, respectively, concerning to global time combinations for Root_200_ (Fig 3G–3I and S2 Table).

**Fig 4:**
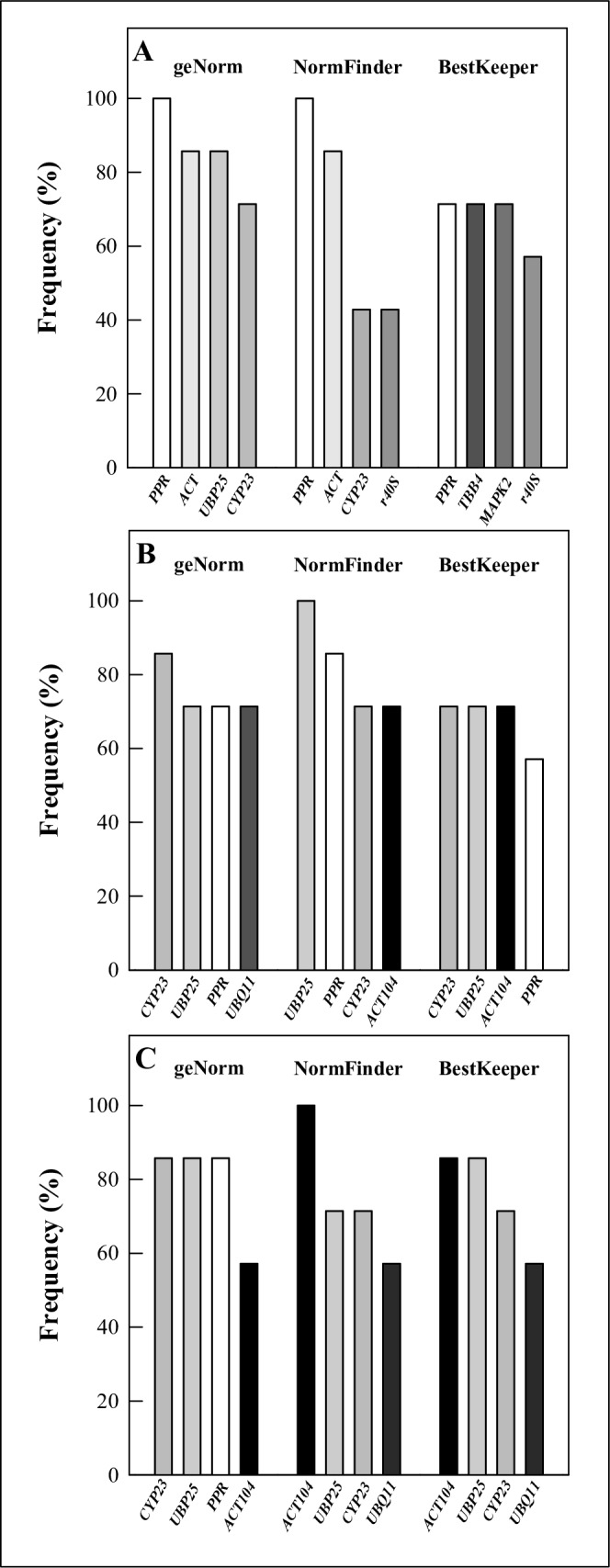
Frequency of the four top-ranked reference genes (RGs) among seven time combinations concerning the first hours (30 min, 2 h, 8 h) of salt stress in geNorm, NormFinder and BestKeeper. Tissues regard *Calotropis procera* leaf and root samples under different salt stress time points: A) Leaf_100_ (100 mM NaCl), B) Root_50_ (50 mM NaCl) and C) Root_200_ (200 mM NaCl).

Isolating the first hours (30 min, 2 h, 8 h) for each experiment and their factorial combinations (totaling seven time combinations), revealed the more frequent RGs among the rank top four out of seven time combinations (Fig 4 and S2 Table). For Leaf_100_, the more frequent RG was *PPR*, according to geNorm and NormFinder. *PPR*, *TBB4* and *MAPK2*, according to BestKeeper (Fig 4A and S2 Table). For Root_50,_ the more frequent RGs was *CYP23*, according to geNorm; *UBP25*, according to NormFinder; *CYP23*, *UBP25* and *ACT104*, according to BestKeeper (Fig 4B and S2 Table). For Root_200_, the more frequent RGs were *CYP23*, *UBP25* and *PPR* according to geNorm; *ACT104* according to NormFinder; *ACT104* and *UBP25* according to BestKeeper (Fig 4C and S2 Table).

### Target genes expression in different experimental conditions by qPCR

The transcriptional patterns of three TGs (*ND1*, *CNBL4*, and *NAC78*) under Leaf_100_ (2 h, 8 h, and 45 days) and Root_200_ (2 h, 8 h and 1 day) were analyzed using the most suitable reference genes for Leaf_100_ (*CYP23* and *ACT*) and Root_200_ (*CYP23*, *UBP25* and *ACT104*) as recommended by geNorm (S2 Table).

**Fig 5:**
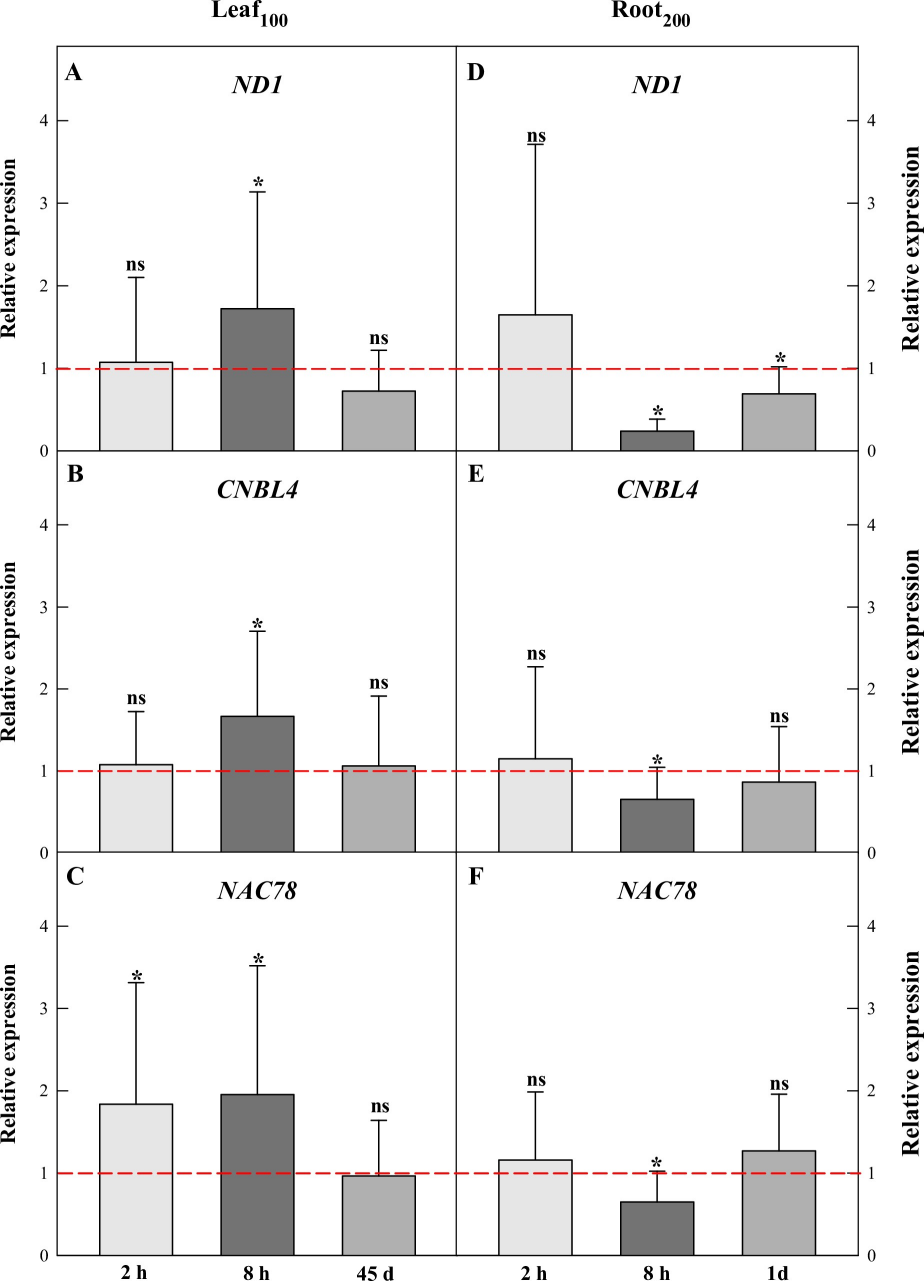
Relative expression of the target genes *ND1*, *CNBL4* and *NAC78* in *Calotropis procera* Leaf_200_ (A, B, C) and Root_200_ experiments (D, E, F). The references genes used were *CYP23* and *ACT* (in Leaf_100_) *CYP23*, *UBP25* and *ACT104* (in Root_200_). Leaf_100_ and Root_200:_ salt stress by concentrations of NaCl 100 and 200 mM, respectively. Values followed by * means P < 0.05. Up-regulation of gene expression (up); down-regulation of gene expression (down); ns (not significant at p < 0.05, or constitutive expression); relative expression values below or above the red line, associated with ‘*’, indicate up- and down-regulation, respectively.

In short-term salt stress (2 h), gene expression analysis via qPCR revealed that most target genes exhibited constitutive expression in both salt-stressed tissues (Leaf_100_ and Root_200_) (Fig 5). The only exception was *NAC78*, which was up-regulated in Leaf_100_ (Fig 5C). Interestingly, the gene expression modulation occurred, preferentially, at 8 h of salt-stress, with up-regulation in Leaf_100_ (Fig 5A–5C) and down-regulation in Root_200_ (Fig 5D–5F) of all TGs tested. On the other hand, in the last treatment times after salt stress (i.e., 45 days and 1 day, in Leaf_100_ and Root_200_, respectively) we observed constitutive expression for three target genes. The exception occurred for *ND1* at 1 day (in Root_200_), in which the expression was down-regulated (Fig 5D).

Additionally, we compared the qPCR/ Leaf_100_ relative expression and Leaf_100_ RNA-Seq data. Our results revealed that *CNBL4*, *NAC78* qPCR results in Leaf_100_ 2 h, 8 h, and 45 d (Fig 5B and 5C) were according to the respective RNA-Seq data (Table 1). For *ND1*, the qPCR (Fig 5A) and RNA-Seq (Table 1) gene expression results converged in the 2 h and 45 days treatments.

## Discussion

The advent of high-throughput next-generation DNA sequencing (NGS) platforms has provided more comprehensive and maximized studies on diverse genomes, including non-model plant species [7,43,44]. At the same time, advances in RNA sequencing (RNA-Seq) methods have effectively aided in characterization and quantification of transcriptomes (even without a reference genome). They contributed to the understanding of genes expression regulation under different experimental conditions [6,45,46]. However, due to the existence of potential errors during the preparation, synthesis, sequencing and analysis of gene expression libraries (including RNA-Seq), a second method is required to validate the results indicated by the first. The qPCR is currently the most appropriated method for such purpose [12,47], and quality control measures are necessary to mitigate potential errors in qPCR results. Thus, the selection of suitable reference genes is a fundamental requisite. The use of inappropriate RGs may overestimate or underestimate the relative expression of the target genes and lead to [18].

In this study, transcripts of *C*. *procera* (RNA-Seq), identified statistically as constitutively expressed (considering log_2_FC and P-value), were used as a source for candidate reference genes screening. The expression levels and stability analysis of ten RGs were evaluated in leaf and root samples of *C*. *procera* under different salt concentrations (NaCl). Using geNorm, NormFinder and BestKeeper software allowed us to analyze the expression stability of RGs in salt concentrations individually and factorial of time combinations.

According to the Cq-value and stability expression analysis, discrepancies were observed among candidate RGs under all conditions studied (including different tissues, salt concentrations and collection time combinations), indicating the importance of studies on RGs stability under different experimental conditions. Although several works have reported the use of traditional RGs as suitable in qPCR assays [48–50], recent studies have shown expression stability for many of these genes may be affected in different plant species under experimental conditions [16,20]. These reports, consistent with our results, support the careful evaluation of candidate RGs under given experimental conditions [16,51].

The RGs stability rankings suggested by different softwares were not often entirely identical for the same experimental conditions, as distinct statistical algorithms and analytical procedures are applied [52]. Despite the high degree of similarities, we found less congruence between results of geNorm *vs*. BestKeeper, for Leaf_100_ (30 min, 2 h, 8 h, and 45 days) (Fig 2A). Such a relative divergence between BestKeeper and other softwares was also reported by other authors. According to Zhang et al.[33], in an experiment conducted on *Halostachys caspica* under salt stress, 25% congruence between BestKeeper *vs*. geNorm and 100% between geNorm *vs*. NormFinder were found. Similarly, de Andrade et al. [53] found high correlation among geNorm *vs*. NormFinder. However, geNorm *vs*. BestKeeper showed the lowest correlation. In this context, the choice of RGs to use in the qPCR analysis was determined by geNorm and confirmed by other softwares. The geNorm is one of the most widely used for gene expression stability analysis, besides informing the optimal number of RGs necessary to validate the TGs [16,17].

In spite of RGs specificity for each time combination, we found *CYP23*, a *Peptidyl-prolyl cis-trans isomerase* involved in key processes of protein folding [54], as the most frequent among the four most stable RGs, considering all experimental conditions studied. Similarly, Singh et al. [32] found *CYP* as the most stable RGs for wounding, heat, methyl jasmonate and biotic stress, for different tissues and combined stress samples. Based on this scenario, *CYP23* is a powerful RG candidate to be further tested on expression analysis of *C*. *procera* under different experimental conditions, especially under salinity.

Analyzing all time combinations on Leaf_100_ experiment, we found *ACT*, a cytoskeletal protein associated with plant cell growth [55], as the most frequent RG among the four most stable RGs. Previously, actins were identified as stable RGs in salt, drought, cold and heat stress [31,52]. Furthermore, *UBP25* was most frequent RG among the four most stable for Root_50_ and Root_200_ experiments. On the other hand, *UBP25* was the less stable for Leaf_100_ submitted to prolonged period of salinity (45 days). Interestingly, all time combinations containing time 45 days for Leaf_100_ showed *UBP25* as one of the less stable RGs, considering all softwares studied. However, *UBP25* was among four most stable RGs for most of the first hours combinations (excluding 45 days) on Leaf_100_. *UBP25* participates in ubiquitin-proteasome system (UPS) for maintenance of homeostasis and modulation of the stability proteins under salinity and other abiotic stresses [56,57], inducing the less stability under the high salt stress (45 days, Leaf_100_) compared to the other candidate RGs.

The following target genes, related to salt-stress response, had their expression analyzed by RNA-Seq and qPCR: *ND1* (*NADH dehydrogenase subunit 1*), which acts on the mitochondrial electron transport chain and is involved with rapid systemic signaling triggered by salinity and other abiotic stresses [34]; *CNBL4* (*Calcineurin B-like protein*) involved on SOS pathway as calcium sensors, working in combination with kinases and ion channels to exclude cytosolic salt [35,36]; and *NAC78* (*NAC domain-containing protein 78-like*) that belongs to *NAC* transcription factor family (*NAC*-TFs) involved in regulating plant growth, development processes and abiotic stress responses, including drought and salinity [37,38]. The qPCR data of *ND1* (exception for 8 h treatment), *CNBL4*, and *NAC78* for Leaf_100_ experiment are in agreement with RNA-Seq expression results. The convergence of the results between these two approaches (that is, data validation) increases the robustness of our gene expression data, since the qPCR is considered a gold standard validation method for expression analysis. The up-regulation of the gene expression in response to salt stress as *CNBL4*, *NAC78* and *ND1* in leaf tissue, contribute to the establishment in *Calotropis procera* to high salinity adverse environments.

Regarding the root expression of target genes (qPCR / Root_200_ experiment), they were not up-regulated in any of the treatments (showing up down-regulation or constitutive expression). When compared to Leaf_100_ experiment results, this suggests: *CNBL4*, *NAC78* and *ND1* participate, more actively, in leaf response to salt stress (that is, tissue-specific transcriptional modulation); and /or the transcriptional modulation of the referred targets is dependent on the NaCl concentration. To determine the cause associated with those results, further inquiries are required. However, this gene sample already suggests the complexity of the molecular physiology of *C*. *procera* under stress, highlighting the capacity of adaptation of its transcriptome to different conditions and/or to the demand of different organs.

Our study provides, powerful background about ten candidate RGs for the first time, which can be used in *C*. *procera* studies under salt stress and can provide great potential to be tested in other experimental conditions. We indicate the most reliable RGs for 15-time combinations under three different experimental conditions, including two plant tissues and three NaCl concentrations. The *CYP23* is a powerful RG candidate for expression normalization of *C*. *procera* under different experimental conditions. In addition, *UBP25* should be avoided as RG for long-lasting salt stress in *C*. *procera’s* leaf. Finally, our findings emphasize the need for caution when evaluating the RGs stability in a set of samples under high amplitude of variant factors. The use of more than one software supported a reliable way to select the best RGs to validate TGs on qPCR.

## Supporting information

S1 Table Experimental conditions/ samples collected for *Calotropis procera* RNA-Seq libraries and qPCR assays. (DOCX)

S2 Table Expression stability rank and pairwise variation of candidate reference genes in 15 time combinations in leaf and root samples of *Calotropis procera* under different salinity conditions [Leaf_100_ (100 mM NaCl), Root_50_ (50 mM NaCl), Root_200_ (200 mM NaCl)] after geNorm, NormFinder and BestKeeper analysis. † SD above 1, genes excluded from the rank of BestKeeper. * Values followed by * variables do not depend linearly on each other are according to the Pearson's correlation test (p < 0.05).
(DOCX)

S1 Fig Experimental designs for stress application and RNA-Seq libraries sequencing performed in the present work. Legend: RB: biological replicate.
(TIF)

S2 Fig Amplification products of 10 candidate reference genes and three target genes in agarose gel (2%) from *Calotropis procera* by PCR. M: marker 100 bp; 1–2 *ND1*; 3–4 *CNBL4*; 5–6 *NAC78*; 7–8 *MAPK2*; 9–10 *CYP23*; 11–12 *ACT104*; 13–14 *TBB4*; 15–16 *UBQ11*; 17–18 *ACT*; 19–20 *r40S*; 21–22 *PPR*; 23–24 *UBP25*; 25–26 *FBOX*. Even numbers mean no template control.
(TIF)

S3 Fig Melting temperature (°C) of 10 candidate reference genes and three target genes from *Calotropis procera* by qPCR. Each line represents the melting curve for each individual replicate.
(TIF)

## Abbreviations

ACT: Actin/actin-like conserved site-containing protein
ACT104: Actin 104
CNBL4: calcineurin B-like protein 4
CYP23: Peptidyl-prolyl cis-trans isomerase 23
FBOX: *F-box* protein PP2-A12
MAPK2: Mitogen-activated protein kinase kinase kinase 2
NAC78: *NAC* domain-containing protein 78-like
ND1: *NADH* dehydrogenase subunit 1
PPR: Putative pentatricopeptide repeat-containing protein
r40S: 40S ribosomal protein S3a
TBB4: Tubulin beta-4-chain
UBQ11: Polyubiquitin 11- like
UBP25: Ubiquitin carboxyl-terminal hydrolase 25
RG: reference gene
TG: target gene
Cq: quantification cycle
