# Supplementary material for: Reference genes selection for Calotropis procera under different salt stress conditions
Source: PLoS One. 2019 Apr 18;14(4):e0215729. doi: 10.1371/journal.pone.0215729 (PMC6472812; doi:10.1371/journal.pone.0215729)
Supplement: S1 Table — (DOCX) [file pone.0215729.s001.docx]

| **Experiment** |  | **Salt stress** |  | **Tissue** |  | **Time point colleted** | **RNA-Seq** | **qPCR** |
| --- | --- | --- | --- | --- | --- | --- | --- | --- |
|  |  |  |  |  |  |  |  |  |
|  |  |  |  | leaf* |  | 30 min, 2 h, 8 h and 45 days (treatments) | yes | yes |
| **Leaf_100_** |  | 100 mM NaCl |  | leaf |  | 30 min, 2 h, 8 h (controls) | no | yes |
|  |  |  |  | leaf* |  | 0 h (control) | yes | no |
|  |  |  |  | leaf* |  | 45 days (control) | yes | yes |
|  |  |  |  |  |  |  |  |  |
| **Root_50_** |  | 50 mM NaCl |  | root |  | 30 min, 2 h, 8 h and 1 day | no | yes |
|  |  |  |  |  |  | (controls x treatments) |  |  |
|  |  |  |  |  |  |  |  |  |
| **Root_200_** |  | 200 mM NaCl |  | root |  | 30 min, 2 h, 8 h and 1 day | no | yes |
|  |  |  |  |  |  | (controls x treatments) |  |  |
|  |  |  |  |  |  |  |  |  |

**Table S1.** Experimental conditions/ samples collected for *C. procera* RNA-Seq libraries and qPCR assays.

* Libraries composed by bulk of the three biological replicates. Were sequenced using Illumina paired-end sequencing technology on Illumina Hi-Seq TM 2500 platform.
