# Supplementary material for: Reference genes selection for Calotropis procera under different salt stress conditions
Source: PLoS One. 2019 Apr 18;14(4):e0215729. doi: 10.1371/journal.pone.0215729 (PMC6472812; doi:10.1371/journal.pone.0215729)
Supplement: S2 Table — † SD above 1, genes excluded from the rank of BestKeeper. * Values followed by * variables do not depend linearly on each other are according to the Pearson's correlation test (p < 0.05). (DOCX) [file pone.0215729.s002.docx]

**Table S2.** Expression stability rank and pairwise variation of candidate reference genes in 15 time combinations in leaf and root samples of *Calotropis procera* under different salinity conditions: Leaf_100_ (100 mM NaCl), Root_50_ (50 mM NaCl), Root_200_ (200 mM NaCl) and using the geNorm, NormFinder and BestKeeper software. Leaf_100:_ leaf samples collected at 30 min, 2 h, 8 h and 45 days after 100 mM NaCl; Root_50_ and Root_200_: root samples collected at 30 min, 2 h, 8 h and 1 day after 50 and 200 mM NaCl, respectively. V, pairwise variation; † SD >1, genes excluded from the rank of BestKeeper. Values followed by ***** variables do not depend linearly on each other are according to the Pearson's correlation test (p < 0.05).

|  |  |  |  | **geNorm** | | | | | | | | | |  |  |  | **NormFinder** | | | | | | | | | |  | **BestKeeper** | | | | | | | | | |
| --- | --- | --- | --- | --- | --- | --- | --- | --- | --- | --- | --- | --- | --- | --- | --- | --- | --- | --- | --- | --- | --- | --- | --- | --- | --- | --- | --- | --- | --- | --- | --- | --- | --- | --- | --- | --- | --- |
|  |  |  |  |  |  |  |  |  |  |  |  |  |  | **V** | |  |  |  |  |  |  |  |  |  |  |  |  |  |  |  |  |  |  |  |  |  |  |
| **Assay** |  | **Time combination** |  | **1** | **2** | **3** | **4** | **5** | **6** | **7** | **8** | **9** | **10** | **2/3** | **3/4** |  | **1** | **2** | **3** | **4** | **5** | **6** | **7** | **8** | **9** | **10** |  | **1** | **2** | **3** | **4** | **5** | **6** | **7** | **8** | **9** | **10** |
|  |  | 30min-2h-8h-45d |  | *CYP23* | *ACT* | *PPR* | *r40S* | *TBB4* | *ACT104* | *MAPK2* | *FBOX* | *UBQ11* | *UBP25* | 0.14 | 0.16 |  | *ACT* | *TBB4* | *PPR* | *r40S* | *CYP23* | *ACT104* | *MAPK2* | *FBOX* | *UBP25* | *UBQ11* |  | *TBB4** | *ACT104** | *r40S** | *ACT** | *MAPK2** | *CYP23** | *PPR** | *UBQ11** | *UBP25** | *FBOX*†* |
|  |  |  |  | 0.368 | 0.368 | 0.418 | 0.547 | 0.601 | 0.644 | 0.687 | 0.769 | 0.871 | 1.012 |  |  |  | 0.303 | 0.304 | 0.314 | 0.343 | 0.355 | 0.428 | 0.449 | 0.540 | 0.592 | 0.623 |  | 0.942 | 0.867 | 0.863 | 0.822 | 0.812 | 0.804 | 0.790 | 0.465 | 0.434 | 0.922 |
|  |  | 30min-2h-8h |  | *ACT* | *PPR* | *CYP23* | *UBP25* | *r40S* | *ACT104* | *TBB4* | *MAPK2* | *FBOX* | *UBQ11* | 0.11 | 0.09 |  | *PPR* | *TBB4* | *ACT* | *r40S* | *CYP23* | *ACT104* | *UBP25* | *MAPK2* | *UBQ11* | *FBOX* |  | *TBB4** | *MAPK2** | *PPR** | *r40S** | *ACT104** | *ACT** | *CYP23** | *UBP25** | *UBQ11* | *FBOX*†* |
|  |  |  |  | 0.286 | 0.286 | 0.337 | 0.371 | 0.495 | 0.560 | 0.591 | 0.629 | 0.717 | 0.792 |  |  |  | 0.244 | 0.306 | 0.310 | 0.312 | 0.343 | 0.373 | 0.401 | 0.422 | 0.612 | 0.615 |  | 0.973 | 0.944 | 0.944 | 0.940 | 0.922 | 0.876 | 0.869 | 0.761 | 0.448 | 0.928 |
|  |  | 30min-2h-45d |  | *CYP23* | *ACT* | *TBB4* | *PPR* | *r40S* | *FBOX* | *ACT104* | *MAPK2* | *UBQ11* | *UBP25* | 0.14 | 0.11 |  | *TBB4* | *ACT* | *PPR* | *CYP23* | *FBOX* | *r40S* | *MAPK2* | *ACT104* | *UBP25* | *UBQ11* |  | *FBOX** | *TBB4** | *CYP23** | *ACT104** | *ACT** | *r40S** | *PPR** | *MAPK2** | *UBQ11* | *UBP25* |
|  |  |  |  | 0.294 | 0.294 | 0.397 | 0.442 | 0.508 | 0.558 | 0.602 | 0.642 | 0.761 | 0.944 |  |  |  | 0.198 | 0.248 | 0.260 | 0.291 | 0.291 | 0.341 | 0.416 | 0.428 | 0.520 | 0.578 |  | 0.850 | 0.779 | 0.713 | 0.709 | 0.625 | 0.536 | 0.492 | 0.474 | 0.457 | 0.390 |
|  |  | 30min-8h-45d |  | *ACT* | *PPR* | *CYP23* | *r40S* | *TBB4* | *ACT104* | *MAPK2* | *FBOX* | *UBQ11* | *UBP25* | 0.13 | 0.17 |  | *ACT* | *r40S* | *TBB4* | *CYP23* | *PPR* | *ACT104* | *MAPK2* | *FBOX* | *UBQ11* | *UBP25* |  | *TBB4** | *ACT104** | *r40S** | *ACT** | *MAPK2** | *CYP23** | *PPR** | *UBQ11** | *UBP25* | *FBOX*†* |
|  |  |  |  | 0.396 | 0.396 | 0.431 | 0.567 | 0.642 | 0.677 | 0.725 | 0.823 | 0.928 | 1.099 |  |  |  | 0.319 | 0.357 | 0.358 | 0.366 | 0.374 | 0.419 | 0.491 | 0.639 | 0.653 | 0.721 |  | 0.942 | 0.892 | 0.887 | 0.841 | 0.832 | 0.831 | 0.802 | 0.532 | 0.426 | 0.938 |
|  |  | 2h-8h-45d |  | *CYP23* | *ACT* | *PPR* | *r40S* | *TBB4* | *MAPK2* | *ACT104* | *FBOX* | *UBQ11* | *UBP25* | 0.15 | 0.16 |  | *ACT* | *TBB4* | *r40S* | *CYP23* | *PPR* | *MAPK2* | *ACT104* | *FBOX* | *UBQ11* | *UBP25* |  | *TBB4** | *r40S** | *ACT104** | *ACT** | *CYP23** | *MAPK2** | *PPR** | *UBQ11** | *UBP25* | *FBOX*†* |
|  |  |  |  | 0.336 | 0.336 | 0.432 | 0.551 | 0.613 | 0.655 | 0.697 | 0.814 | 0.906 | 1.070 |  |  |  | 0.311 | 0.333 | 0.337 | 0.349 | 0.350 | 0.403 | 0.439 | 0.592 | 0.600 | 0.648 |  | 0.931 | 0.864 | 0.856 | 0.832 | 0.790 | 0.773 | 0.737 | 0.488 | 0.450 | 0.907 |
|  |  | 30min-2h |  | *ACT* | *PPR* | *CYP23* | *TBB4* | *UBP25* | *r40S* | *MAPK2* | *FBOX* | *ACT104* | *UBQ11* | 0.10 | 0.07 |  | *TBB4* | *PPR* | *CYP23* | *ACT* | *UBP25* | *r40S* | *FBOX* | *MAPK2* | *ACT104* | *UBQ11* |  | *TBB4** | *FBOX** | *MAPK2** | *CYP23** | *PPR** | *ACT104** | *UBP25** | *ACT** | *r40S** | *UBQ11* |
|  |  |  |  | 0.256 | 0.256 | 0.299 | 0.314 | 0.330 | 0.350 | 0.381 | 0.405 | 0.446 | 0.562 |  |  |  | 0.154 | 0.168 | 0.183 | 0.190 | 0.201 | 0.270 | 0.272 | 0.285 | 0.367 | 0.562 |  | 0.942 | 0.924 | 0.903 | 0.874 | 0.874 | 0.839 | 0.829 | 0.799 | 0.779 | 0.239 |
| **Leaf_100_** |  | 30mim-8h |  | *ACT* | *PPR* | *CYP23* | *UBP25* | *r40S* | *ACT104* | *TBB4* | *MAPK2* | *UBQ11* | *FBOX* | 0.13 | 0.11 |  | *PPR* | *r40S* | *ACT* | *ACT104* | *TBB4* | *CYP23* | *MAPK2* | *UBP25* | *UBQ11* | *FBOX* |  | *PPR** | *MAPK2** | *CYP23** | *ACT** | *UBP25** | *UBQ11* | *TBB4*†* | *ACT104*†* | *r40S*†* | *FBOX*†* |
|  |  |  |  | 0.291 | 0.291 | 0.361 | 0.417 | 0.558 | 0.636 | 0.668 | 0.712 | 0.788 | 0.870 |  |  |  | 0.294 | 0.328 | 0.344 | 0.362 | 0.379 | 0.399 | 0.492 | 0.510 | 0.626 | 0.748 |  | 0.963 | 0.955 | 0.907 | 0.904 | 0.792 | 0.548 | 0.978 | 0.952 | 0.951 | 0.949 |
|  |  | 30min-45d |  | *CYP23* | *ACT* | *PPR* | *ACT104* | *TBB4* | *r40S* | *FBOX* | *MAPK2* | *UBQ11* | *UBP25* | 0.14 | 0.11 |  | *TBB4* | *PPR* | *ACT* | *FBOX* | *CYP23* | *ACT104* | *r40S* | *MAPK2* | *UBQ11* | *UBP25* |  | *FBOX** | *TBB4** | *CYP23* | *r40s* | *ACT104* | *MAPK2* | *PPR* | *ACT* | *UBP25* | *UBQ11*†* |
|  |  |  |  | 0.337 | 0.337 | 0.409 | 0.450 | 0.489 | 0.560 | 0.618 | 0.677 | 0.793 | 1.040 |  |  |  | 0.203 | 0.259 | 0.287 | 0.292 | 0.316 | 0.356 | 0.360 | 0.464 | 0.549 | 0.691 |  | 0.842 | 0.673 | 0.557 | 0.534 | 0.508 | 0.446 | 0.438 | 0.400 | 0.330 | 0.750 |
|  |  | 2h-8h |  | *ACT* | *UBP25* | *PPR* | *CYP23* | *ACT104* | *r40S* | *TBB4* | *MAPK2* | *UBQ11* | *FBOX* | 0.09 | 0.09 |  | *PPR* | *ACT104* | *r40S* | *ACT* | *TBB4* | *MAPK2* | *CYP23* | *UBP25* | *UBQ11* | *FBOX* |  | *TBB4** | *r40S** | *PPR** | *ACT104** | *MAPK2** | *ACT** | *CYP23** | *UBP25** | *UBQ11* | *FBOX*†* |
|  |  |  |  | 0.286 | 0.286 | 0.300 | 0.347 | 0.410 | 0.493 | 0.537 | 0.581 | 0.653 | 0.758 |  |  |  | 0.217 | 0.254 | 0.291 | 0.324 | 0.326 | 0.357 | 0.366 | 0.381 | 0.530 | 0.646 |  | 0.970 | 0.955 | 0.942 | 0.932 | 0.923 | 0.869 | 0.834 | 0.786 | 0.538 | 0.889 |
|  |  | 2h-45d |  | *CYP23* | *ACT* | *TBB4* | *PPR* | *r40S* | *FBOX* | *MAPK2* | *ACT104* | *UBQ11* | *UBP25* | 0.15 | 0.13 |  | *TBB4* | *ACT* | *PPR* | *FBOX* | *CYP23* | *r40S* | *MAPK2* | *ACT104* | *UBQ11* | *UBP25* |  | *FBOX** | *ACT104** | *CYP23** | *TBB4** | *ACT** | *UBQ11* | *UBP25* | *r40S* | *PPR* | *MAPK2* |
|  |  |  |  | 0.174 | 0.174 | 0.371 | 0.465 | 0.534 | 0.595 | 0.636 | 0.688 | 0.811 | 1.047 |  |  |  | 0.231 | 0.248 | 0.306 | 0.308 | 0.315 | 0.361 | 0.419 | 0.486 | 0.601 | 0.661 |  | 0.739 | 0.694 | 0.680 | 0.638 | 0.612 | 0.426 | 0.421 | 0.328 | 0.089 | 0.026 |
|  |  | 8h-45d |  | *ACT* | *PPR* | *CYP23* | *r40S* | *MAPK2* | *TBB4* | *ACT104* | *UBQ11* | *FBOX* | *UBP25* | 0.14 | 0.14 |  | *r40S* | *CYP23* | *ACT* | *MAPK2* | *TBB4* | *PPR* | *ACT104* | *UBQ11* | *FBOX* | *UBP25* |  | *r40S** | *ACT** | *CYP23** | *MAPK2** | *PPR** | *UBQ11** | *UBP25†* | *TBB4*†* | *FBOX*†* | *ACT104*†* |
|  |  |  |  | 0.390 | 0.390 | 0.436 | 0.517 | 0.602 | 0.656 | 0.706 | 0.846 | 0.965 | 1.180 |  |  |  | 0.283 | 0.293 | 0.308 | 0.391 | 0.402 | 0.449 | 0.471 | 0.605 | 0.673 | 0.752 |  | 0.919 | 0.876 | 0.849 | 0.829 | 0.750 | 0.560 | 0.462 | 0.936 | 0.927 | 0.892 |
|  |  | 30min |  | *TBB4* | *PPR* | *UBP25* | *CYP23* | *ACT* | *r40S* | *MAPK2* | *FBOX* | *ACT104* | *UBQ11* | 0.09 | 0.07 |  | *TBB4* | *PPR* | *UBP25* | *CYP23* | *ACT104* | *MAPK2* | *FBOX* | *ACT* | *r40S* | *UBQ11* |  | *FBOX** | *TBB4** | *UBP25** | *MAPK2** | *PPR** | *CYP23** | *ACT104* | *UBQ11* | *r40S* | *ACT* |
|  |  |  |  | 0.261 | 0.261 | 0.288 | 0.310 | 0.342 | 0.369 | 0.406 | 0.434 | 0.453 | 0.538 |  |  |  | 0.110 | 0.183 | 0.188 | 0.204 | 0.237 | 0.240 | 0.251 | 0.270 | 0.282 | 0.491 |  | 0.979 | 0.942 | 0.916 | 0.907 | 0.896 | 0.860 | 0.790 | 0.712 | 0.711 | 0.702 |
|  |  | 2h |  | *PPR* | *UBP25* | *ACT* | *ACT104* | *CYP23* | *TBB4* | *r40S* | *MAPK2* | *FBOX* | *UBQ11* | 0.08 | 0.05 |  | *ACT* | *ACT104* | *PPR* | *CYP23* | *TBB4* | *UBP25* | *r40S* | *FBOX* | *MAPK2* | *UBQ11* |  | *UBP25** | *r40S** | *PPR** | *MAPK2** | *TBB4* | *CYP23* | *FBOX* | *ACT104* | *ACT* | *UBQ11* |
|  |  |  |  | 0.141 | 0.141 | 0.218 | 0.228 | 0.243 | 0.260 | 0.284 | 0.303 | 0.337 | 0.466 |  |  |  | 0.092 | 0.133 | 0.148 | 0.156 | 0.164 | 0.181 | 0.250 | 0.280 | 0.300 | 0.571 |  | 0.894 | 0.879 | 0.867 | 0.830 | 0.712 | 0.586 | 0.549 | 0.531 | 0.531 | -0.781 |
|  |  | 8h |  | *PPR* | *UBP25* | *ACT* | *r40S* | *TBB4* | *ACT104* | *MAPK2* | *CYP23* | *UBQ11* | *FBOX* | 0.10 | 0.11 |  | *PPR* | *UBP25* | *ACT* | *MAPK2* | *ACT104* | *r40S* | *TBB4* | *CYP23* | *UBQ11* | *FBOX* |  | *PPR** | *TBB4** | *UBP25** | *r40S** | *ACT** | *ACT104** | *MAPK2** | *CYP23** | *UBQ11* | *FBOX†* |
|  |  |  |  | 0.272 | 0.272 | 0.302 | 0.391 | 0.439 | 0.470 | 0.495 | 0.536 | 0.595 | 0.655 |  |  |  | 0.100 | 0.126 | 0.130 | 0.167 | 0.175 | 0.175 | 0.197 | 0.235 | 0.295 | 0.304 |  | 0.984 | 0.974 | 0.970 | 0.962 | 0.958 | 0.949 | 0.948 | 0.896 | 0.730 | 0.912 |
|  |  | 45d |  | *CYP23* | *ACT* | *PPR* | *ACT104* | *r40S* | *TBB4* | *MAPK2* | *FBOX* | *UBQ11* | *UBP25* | 0.06 | 0.10 |  | *r40S* | *ACT* | *CYP23* | *TBB4* | *PPR* | *FBOX* | *ACT104* | *MAPK2* | *UBQ11* | *UBP25* |  | *r40S* | *FBOX* | *TBB4* | *ACT104* | *CYP23* | *MAPK2* | *ACT* | *PPR* | *UBQ11*†* | *UBP25†* |
|  |  |  |  | 0.114 | 0.114 | 0.165 | 0.296 | 0.438 | 0.497 | 0.598 | 0.673 | 0.841 | 1.240 |  |  |  | 0.210 | 0.265 | 0.269 | 0.292 | 0.326 | 0.333 | 0.411 | 0.449 | 0.583 | 1.150 |  | 0.713 | 0.641 | 0.322 | 0.269 | 0.197 | 0.121 | 0.095 | -0.020 | 0.885 | 0.304 |
|  |  | 30min-2h-8h-1d |  | *CYP23* | *UBP25* | *ACT104* | *ACT* | *PPR* | *UBQ11* | *MAPK2* | *r40S* | *TBB4* | *FBOX* | 0.23 | 0.14 |  | *CYP23* | *UBP25* | *ACT104* | *UBQ11* | *PPR* | *ACT* | *MAPK2* | *r40S* | *TBB4* | *FBOX* |  | *ACT104** | *CYP23** | *UBP25** | *ACT** | *UBQ11** | *PPR** | *MAPK2** | *TBB4*†* | *r40S*†* | *FBOX*†* |
|  |  |  |  | 0.502 | 0.502 | 0.655 | 0.666 | 0.725 | 0.754 | 0.847 | 0.940 | 1.008 | 1.133 |  |  |  | 0.273 | 0.277 | 0.324 | 0.444 | 0.456 | 0.465 | 0.606 | 0.622 | 0.699 | 0.827 |  | 0.943 | 0.910 | 0.893 | 0.874 | 0.726 | 0.638 | 0.404 | 0.866 | 0.755 | 0.577 |
|  |  | 30min-2h-8h |  | *CYP23* | *PPR* | *UBP25* | *UBQ11* | *ACT104* | *ACT* | *TBB4* | *MAPK2* | *r40S* | *FBOX* | 0.17 | 0.14 |  | *CYP23* | *PPR* | *UBP25* | *ACT104* | *UBQ11* | *ACT* | *r40S* | *MAPK2* | *TBB4* | *FBOX* |  | *ACT104** | *CYP23** | *UBP25** | *PPR** | *r40S** | *UBQ11** | *FBOX** | *MAPK2* | *TBB4*†* | *ACT*†* |
|  |  |  |  | 0.440 | 0.440 | 0.519 | 0.577 | 0.704 | 0.752 | 0.856 | 0.943 | 1.010 | 1.122 |  |  |  | 0.304 | 0.341 | 0.353 | 0.388 | 0.448 | 0.578 | 0.586 | 0.645 | 0.672 | 0.808 |  | 0.967 | 0.880 | 0.841 | 0.804 | 0.718 | 0.647 | 0.468 | 0.225 | 0.885 | 0.874 |
|  |  | 30min-2h-1d |  | *ACT104* | *ACT* | *UBP25* | *CYP23* | *MAPK2* | *UBQ11* | *PPR* | *r40S* | *TBB4* | *FBOX* | 0.25 | 0.16 |  | *CYP23* | *UBP25* | *ACT104* | *ACT* | *MAPK2* | *PPR* | *UBQ11* | *R40s* | *TBB4* | *FBOX* |  | *ACT104** | *ACT** | *CYP23** | *UBP25** | *r40S** | *MAPK2** | *UBQ11** | *PPR** | *TBB4*†* | *FBOX*†* |
|  |  |  |  | 0.358 | 0.358 | 0.623 | 0.678 | 0.757 | 0.799 | 0.822 | 0.907 | 0.982 | 1.072 |  |  |  | 0.267 | 0.274 | 0.358 | 0.388 | 0.439 | 0.459 | 0.517 | 0.570 | 0.664 | 0.716 |  | 0.945 | 0.934 | 0.908 | 0.894 | 0.720 | 0.695 | 0.663 | 0.610 | 0.854 | 0.673 |
|  |  | 30min-8h-1d |  | *ACT104* | *UBP25* | *CYP23* | *UBQ11* | *ACT* | *PPR* | *MAPK2* | *r40S* | *TBB4* | *FBOX* | 0.17 | 0.13 |  | *UBP25* | *ACT104* | *CYP23* | *UBQ11* | *ACT* | *PPR* | *MAPK2* | *r40S* | *TBB4* | *FBOX* |  | *ACT104** | *UBP25** | *CYP23** | *UBQ11** | *ACT** | *r40S** | *PPR** | *MAPK2* | *TBB4*†* | *FBOX†* |
|  |  |  |  | 0.515 | 0.515 | 0.543 | 0.569 | 0.616 | 0.657 | 0.768 | 0.868 | 0.971 | 1.115 |  |  |  | 0.237 | 0.258 | 0.258 | 0.314 | 0.451 | 0.464 | 0.616 | 0.616 | 0.777 | 0.908 |  | 0.936 | 0.918 | 0.904 | 0.877 | 0.820 | 0.666 | 0.561 | 0.423 | 0.845 | 0.438 |
|  |  | 2h-8h-1d |  | *CYP23* | *UBP25* | *ACT104* | *ACT* | *UBQ11* | *PPR* | *TBB4* | *r40S* | *MAPK2* | *FBOX* | 0.23 | 0.15 |  | *CYP23* | *UBP25* | *ACT104* | *ACT* | *UBQ11* | *PPR* | *r40S* | *TBB4* | *MAPK2* | *FBOX* |  | *CYP23** | *ACT104** | *UBP25** | *ACT** | *UBQ11** | *PPR** | *MAPK2* | *TBB4*†* | *r40S*†* | *FBOX*†* |
|  |  |  |  | 0.386 | 0.386 | 0.602 | 0.652 | 0.734 | 0.786 | 0.875 | 0.936 | 1.013 | 1.158 |  |  |  | 0.240 | 0.245 | 0.295 | 0.444 | 0.475 | 0.534 | 0.628 | 0.631 | 0.696 | 0.852 |  | 0.945 | 0.943 | 0.913 | 0.862 | 0.723 | 0.601 | 0.390 | 0.887 | 0.856 | 0.671 |
|  |  | 30min-2h |  | *CYP23* | *PPR* | *MAPK2* | *UBP25* | *UBQ11* | *ACT* | *ACT104* | *r40S* | *FBOX* | *TBB4* | 0.18 | 0.16 |  | *PPR* | *CYP23* | *UBP25* | *ACT104* | *MAPK2* | *ACT* | *UBQ11* | *r40S* | *FBOX* | *TBB4* |  | *CYP23** | *PPR** | *UBP25** | *r40S** | *FBOX** | *MAPK2* | *UBQ11* | *ACT104*†* | *ACT*†* | *TBB4*†* |
|  |  |  |  | 0.432 | 0.432 | 0.525 | 0.606 | 0.655 | 0.790 | 0.849 | 0.945 | 1.003 | 1.067 |  |  |  | 0.301 | 0.313 | 0.389 | 0.477 | 0.497 | 0.522 | 0.573 | 0.645 | 0.665 | 0.674 |  | 0.900 | 0.889 | 0.835 | 0.681 | 0.637 | 0.543 | 0.520 | 0.982 | 0.961 | 0.888 |
| **Root_50_** |  | 30min-8h |  | *CYP23* | *PPR* | *UBQ11* | *UBP25* | *ACT104* | *ACT* | *r40S* | *MAPK2* | *TBB4* | *FBOX* | 0.18 | 0.12 |  | *UBQ11* | *PPR* | *ACT104* | *UBP25* | *CYP23* | *r40S* | *ACT* | *MAPK2* | *TBB4* | *FBOX* |  | *ACT104** | *UBQ11** | *UBP25** | *ACT** | *PPR** | *CYP23** | *r40S* | *MAPK2* | *FBOX* | *TBB4*†* |
|  |  |  |  | 0.379 | 0.379 | 0.515 | 0.536 | 0.553 | 0.624 | 0.738 | 0.831 | 0.924 | 1.069 |  |  |  | 0.275 | 0.299 | 0.306 | 0.326 | 0.329 | 0.526 | 0.613 | 0.638 | 0.718 | 0.938 |  | 0.960 | 0.943 | 0.867 | 0.779 | 0.775 | 0.768 | 0.396 | 0.255 | -0.128 | 0.876 |
|  |  | 30min-1d |  | *ACT104* | *ACT* | *UBP25* | *CYP23* | *MAPK2* | *UBQ11* | *PPR* | *r40S* | *TBB4* | *FBOX* | 0.17 | 0.16 |  | *UBP25* | *CYP23* | *ACT104* | *ACT* | *MAPK2* | *UBQ11* | *PPR* | *r40S* | *TBB4* | *FBOX* |  | *UBP25** | *ACT104** | *ACT** | *CYP23** | *UBQ11** | *MAPK2** | *r40S* | *PPR* | *TBB4*†* | *FBOX†* |
|  |  |  |  | 0.295 | 0.295 | 0.445 | 0.568 | 0.610 | 0.648 | 0.697 | 0.791 | 0.922 | 1.037 |  |  |  | 0.221 | 0.233 | 0.284 | 0.327 | 0.364 | 0.388 | 0.488 | 0.530 | 0.755 | 0.787 |  | 0.922 | 0.919 | 0.910 | 0.889 | 0.838 | 0.806 | 0.504 | 0.455 | 0.808 | 0.558 |
|  |  | 2h-8h |  | *CYP23* | *UBP25* | *PPR* | *UBQ11* | *ACT104* | *ACT* | *TBB4* | *r40S* | *MAPK2* | *FBOX* | 0.16 | 0.15 |  | *CYP23* | *UBP25* | *ACT104* | *PPR* | *UBQ11* | *r40S* | *TBB4* | *ACT* | *MAPK2* | *FBOX* |  | *ACT104** | *CYP23** | *r40S** | *UBP25** | *ACT** | *PPR** | *UBQ11* | *MAPK2* | *TBB4*†* | *FBOX*†* |
|  |  |  |  | 0.449 | 0.449 | 0.499 | 0.575 | 0.736 | 0.809 | 0.880 | 0.956 | 1.041 | 1.171 |  |  |  | 0.269 | 0.343 | 0.377 | 0.450 | 0.510 | 0.556 | 0.570 | 0.608 | 0.760 | 0.845 |  | 0.964 | 0.935 | 0.871 | 0.847 | 0.843 | 0.769 | 0.554 | 0.211 | 0.914 | 0.626 |
|  |  | 2h-1d |  | *CYP23* | *UBP25* | *ACT* | *ACT104* | *TBB4* | *r40S* | *MAPK2* | *UBQ11* | *PPR* | *FBOX* | 0.26 | 0.15 |  | *UBP25* | *CYP23* | *ACT* | *ACT104* | *MAPK2* | *r40S* | *TBB4* | *UBQ11* | *PPR* | *FBOX* |  | *CYP23** | *ACT104** | *ACT** | *UBP25** | *MAPK2** | *UBQ11** | *PPR* | *TBB4*†* | *r40S*†* | *FBOX*†* |
|  |  |  |  | 0.378 | 0.378 | 0.655 | 0.674 | 0.720 | 0.778 | 0.855 | 0.914 | 0.953 | 1.062 |  |  |  | 0.223 | 0.223 | 0.315 | 0.343 | 0.465 | 0.489 | 0.515 | 0.541 | 0.563 | 0.690 |  | 0.953 | 0.949 | 0.938 | 0.928 | 0.713 | 0.660 | 0.551 | 0.898 | 0.856 | 0.804 |
|  |  | 8h-1d |  | *CYP23* | *UBP25* | *ACT104* | *UBQ11* | *ACT* | *PPR* | *r40S* | *TBB4* | *MAPK2* | *FBOX* | 0.11 | 0.12 |  | *UBP25* | *ACT104* | *CYP23* | *UBQ11* | *ACT* | *PPR* | *r40S* | *TBB4* | *MAPK2* | *FBOX* |  | *CYP23** | *ACT104** | *UBP25** | *UBQ11** | *ACT** | *PPR* | *MAPK2* | *TBB4*†* | *r40S*†* | *FBOX†* |
|  |  |  |  | 0.334 | 0.334 | 0.356 | 0.443 | 0.529 | 0.619 | 0.759 | 0.843 | 0.931 | 1.120 |  |  |  | 0.166 | 0.170 | 0.198 | 0.317 | 0.415 | 0.530 | 0.623 | 0.704 | 0.731 | 1.007 |  | 0.964 | 0.963 | 0.947 | 0.911 | 0.786 | 0.508 | 0.426 | 0.848 | 0.825 | 0.570 |
|  |  | 30min |  | *MAPK2* | *CYP23* | *PPR* | *UBQ11* | *UBP25* | *ACT104* | *ACT* | *r40S* | *FBOX* | *TBB4* | 0.16 | 0.14 |  | *PPR* | *UBQ11* | *CYP23* | *UBP25* | *MAPK2* | *ACT104* | *ACT* | *r40S* | *FBOX* | *TBB4* |  | *ACT104** | *ACT** | *PPR** | *UBQ11** | *UBP25** | *CYP23* | *MAPK2* | *r40S* | *FBOX* | *TBB4*†* |
|  |  |  |  | 0.392 | 0.392 | 0.462 | 0.541 | 0.609 | 0.664 | 0.706 | 0.798 | 0.882 | 1.025 |  |  |  | 0.137 | 0.313 | 0.381 | 0.388 | 0.401 | 0.421 | 0.544 | 0.682 | 0.806 | 0.906 |  | 0.974 | 0.971 | 0.910 | 0.904 | 0.864 | 0.795 | 0.638 | -0.429 | -0.708 | 0.938 |
|  |  | 2h |  | *ACT104* | *TBB4* | *ACT* | *FBOX* | *CYP23* | *UBP25* | *PPR* | *r40S* | *MAPK2* | *UBQ11* | 0.16 | 0.20 |  | *CYP23* | *UBP25* | *PPR* | *TBB4* | *ACT* | *ACT104* | *FBOX* | *MAPK2* | *r40S* | *UBQ11* |  | *CYP23** | *PPR** | *UBP25** | *MAPK2* | *UBQ11* | *ACT104*†* | *TBB4*†* | *ACT*†* | *FBOX*†* | *r40S*†* |
|  |  |  |  | 0.460 | 0.460 | 0.498 | 0.663 | 0.808 | 0.879 | 0.905 | 0.951 | 1.025 | 1.091 |  |  |  | 0.255 | 0.406 | 0.443 | 0.502 | 0.516 | 0.543 | 0.556 | 0.647 | 0.676 | 0.815 |  | 0.993 | 0.905 | 0.877 | 0.527 | 0.337 | 0.990 | 0.964 | 0.962 | 0.955 | 0.898 |
|  |  | 8h |  | *ACT104* | *UBQ11* | *CYP23* | *UBP25* | *PPR* | *MAPK2* | *r40S* | *TBB4* | *ACT* | *FBOX* | 0.13 | 0.10 |  | *UBQ11* | *ACT104* | *MAPK2* | *UBP25* | *CYP23* | *r40S* | *PPR* | *TBB4* | *ACT* | *FBOX* |  | *UBQ11** | *ACT104** | *MAPK2** | *CYP23** | *UBP25** | *TBB4* | *r40S* | *PPR* | *ACT* | *FBOX†* |
|  |  |  |  | 0.257 | 0.257 | 0.349 | 0.404 | 0.464 | 0.537 | 0.608 | 0.680 | 0.752 | 1.001 |  |  |  | 0.162 | 0.194 | 0.270 | 0.278 | 0.284 | 0.395 | 0.438 | 0.580 | 0.716 | 1.173 |  | 0.974 | 0.956 | 0.855 | 0.835 | 0.818 | 0.802 | 0.709 | 0.529 | 0.461 | -0.160 |
|  |  | 1d |  | *ACT* | *UBP25* | *CYP23* | *ACT104* | *MAPK2* | *r40S* | *UBQ11* | *TBB4* | *PPR* | *FBOX* | 0.09 | 0.08 |  | *UBP25* | *CYP23* | *ACT104* | *ACT* | *r40S* | *MAPK2* | *UBQ11* | *TBB4* | *PPR* | *FBOX* |  | *UBP25** | *CYP23** | *ACT** | *ACT104** | *UBQ11** | *r40S** | *MAPK2** | *TBB4* | *PPR* | *FBOX*†* |
|  |  |  |  | 0.176 | 0.176 | 0.232 | 0.291 | 0.355 | 0.413 | 0.481 | 0.545 | 0.618 | 0.834 |  |  |  | 0.057 | 0.077 | 0.123 | 0.136 | 0.245 | 0.266 | 0.305 | 0.361 | 0.417 | 0.702 |  | 0.991 | 0.985 | 0.979 | 0.970 | 0.955 | 0.901 | 0.890 | 0.783 | 0.365 | 0.839 |
|  |  | 30min-2h--8h-1d |  | *CYP23* | *UBP25* | *ACT104* | *ACT* | *PPR* | *UBQ11* | *r40S* | *MAPK2* | *TBB4* | *FBOX* | 0.20 | 0.14 |  | *UBP25* | *CYP23* | *ACT104* | *r40S* | *ACT* | *UBQ11* | *PPR* | *MAPK2* | *TBB4* | *FBOX* |  | *ACT104** | *ACT** | *UBP25** | *CYP23** | *r40S** | *PPR** | *UBQ11** | *MAPK2* | *TBB4*†* | *FBOX*†* |
|  |  |  |  | 0.452 | 0.452 | 0.570 | 0.596 | 0.651 | 0.687 | 0.751 | 0.824 | 0.911 | 1.039 |  |  |  | 0.248 | 0.301 | 0.342 | 0.427 | 0.462 | 0.486 | 0.489 | 0.530 | 0.651 | 0.820 |  | 0.950 | 0.933 | 0.904 | 0.866 | 0.776 | 0.747 | 0.724 | 0.260 | 0.697 | 0.543 |
|  |  | 30min-2h-8h |  | *CYP23* | *PPR* | *UBP25* | *ACT104* | *ACT* | *UBQ11* | *r40S* | *MAPK2* | *TBB4* | *FBOX* | 0.17 | 0.16 |  | *UBP25* | *CYP23* | *ACT104* | *r40S* | *PPR* | *UBQ11* | *ACT* | *MAPK2* | *TBB4* | *FBOX* |  | *ACT104** | *UBP25** | *CYP23** | *PPR** | *r40S** | *UBQ11** | *FBOX** | *MAPK2* | *ACT*†* | *TBB4*†* |
|  |  |  |  | 0.354 | 0.354 | 0.477 | 0.580 | 0.635 | 0.686 | 0.756 | 0.853 | 0.946 | 1.050 |  |  |  | 0.280 | 0.324 | 0.335 | 0.432 | 0.466 | 0.496 | 0.520 | 0.583 | 0.690 | 0.819 |  | 0.973 | 0.893 | 0.869 | 0.797 | 0.778 | 0.710 | 0.468 | 0.152 | 0.936 | 0.777 |
|  |  | 30min-2h-1d |  | *ACT104* | *ACT* | *UBP25* | *CYP23* | *PPR* | *UBQ11* | *MAPK2* | *r40S* | *TBB4* | *FBOX* | 0.22 | 0.14 |  | *UBP25* | *ACT104* | *CYP23* | *ACT* | *MAPK2* | *r40S* | *PPR* | *UBQ11* | *TBB4* | *FBOX* |  | *ACT104** | *ACT** | *UBP25** | *CYP23** | *r40S** | *PPR** | *UBQ11** | *TBB4** | *MAPK2** | *FBOX*†* |
|  |  |  |  | 0.383 | 0.383 | 0.588 | 0.616 | 0.668 | 0.709 | 0.765 | 0.829 | 0.925 | 1.048 |  |  |  | 0.255 | 0.304 | 0.318 | 0.421 | 0.446 | 0.458 | 0.486 | 0.542 | 0.621 | 0.762 |  | 0.940 | 0.922 | 0.897 | 0.848 | 0.729 | 0.729 | 0.714 | 0.637 | 0.563 | 0.695 |
|  |  | 30min-8h-1d |  | *ACT104* | *ACT* | *CYP23* | *UBP25* | *UBQ11* | *PPR* | *r40S* | *MAPK2* | *TBB4* | *FBOX* | 0.20 | 0.13 |  | *UBP25* | *CYP23* | *ACT104* | *r40S* | *ACT* | *UBQ11* | *PPR* | *MAPK2* | *TBB4* | *FBOX* |  | *ACT** | *ACT104** | *UBP25** | *CYP23** | *UBQ11** | *PPR** | *r40S** | *TBB4** | *FBOX* | *MAPK2* |
|  |  |  |  | 0.356 | 0.356 | 0.537 | 0.568 | 0.612 | 0.638 | 0.700 | 0.781 | 0.896 | 1.011 |  |  |  | 0.242 | 0.254 | 0.341 | 0.399 | 0.417 | 0.419 | 0.476 | 0.524 | 0.730 | 0.732 |  | 0.943 | 0.915 | 0.907 | 0.863 | 0.846 | 0.749 | 0.629 | 0.490 | 0.333 | 0.147 |
|  |  | 2h-8h-1d |  | *CYP23* | *UBP25* | *ACT104* | *ACT* | *r40S* | *UBQ11* | *PPR* | *TBB4* | *MAPK2* | *FBOX* | 0.20 | 0.14 |  | *UBP25* | *CYP23* | *ACT104* | *r40S* | *UBQ11* | *ACT* | *PPR* | *TBB4* | *MAPK2* | *FBOX* |  | *ACT104** | *UBP25** | *ACT** | *r40S** | *CYP23** | *TBB4** | *UBQ11** | *PPR** | *MAPK2* | *FBOX*†* |
|  |  |  |  | 0.354 | 0.354 | 0.534 | 0.584 | 0.622 | 0.669 | 0.707 | 0.745 | 0.817 | 0.987 |  |  |  | 0.201 | 0.290 | 0.343 | 0.391 | 0.424 | 0.445 | 0.506 | 0.547 | 0.565 | 0.922 |  | 0.954 | 0.933 | 0.929 | 0.921 | 0.885 | 0.840 | 0.771 | 0.702 | 0.227 | 0.618 |
|  |  | 30min-2h |  | *CYP23* | *PPR* | *UBP25* | *ACT104* | *ACT* | *UBQ11* | *MAPK2* | *r40S* | *TBB4* | *FBOX* | 0.16 | 0.18 |  | *ACT104* | *UBP25* | *CYP23* | *ACT* | *MAPK2* | *PPR* | *r40S* | *UBQ11* | *TBB4* | *FBOX* |  | *ACT104** | *ACT** | *UBP25** | *CYP23** | *PPR** | *r40S** | *UBQ11** | *MAPK2* | *TBB4*†* | *FBOX*†* |
|  |  |  |  | 0.272 | 0.272 | 0.417 | 0.587 | 0.633 | 0.712 | 0.765 | 0.846 | 0.968 | 1.058 |  |  |  | 0.282 | 0.301 | 0.371 | 0.392 | 0.399 | 0.448 | 0.484 | 0.631 | 0.672 | 0.727 |  | 0.962 | 0.909 | 0.862 | 0.817 | 0.748 | 0.706 | 0.638 | 0.547 | 0.744 | 0.671 |
| **Root_200_** |  | 30min-8h |  | *CYP23* | *PPR* | *UBQ11* | *UBP25* | *ACT104* | *ACT* | *r40S* | *MAPK2* | *FBOX* | *TBB4* | 0.16 | 0.12 |  | *UBP25* | *CYP23* | *ACT104* | *UBQ11* | *r40S* | *PPR* | *ACT* | *MAPK2* | *FBOX* | *TBB4* |  | *ACT** | *ACT104** | *UBQ11** | *UBP25** | *CYP23** | *PPR** | *r40S** | *MAPK2* | *FBOX* | *TBB4*†* |
|  |  |  |  | 0.388 | 0.388 | 0.478 | 0.513 | 0.539 | 0.559 | 0.621 | 0.758 | 0.864 | 0.981 |  |  |  | 0.275 | 0.278 | 0.332 | 0.365 | 0.371 | 0.437 | 0.487 | 0.623 | 0.691 | 0.766 |  | 0.961 | 0.956 | 0.950 | 0.897 | 0.868 | 0.847 | 0.595 | -0.128 | -0.145 | 0.598 |
|  |  | 30min-1d |  | *ACT104* | *ACT* | *UBP25* | *CYP23* | *PPR* | *UBQ11* | *MAPK2* | *r40S* | *FBOX* | *TBB4* | 0.22 | 0.14 |  | *CYP23* | *UBP25* | *ACT104* | *ACT* | *MAPK2* | *r40S* | *PPR* | *UBQ11* | *FBOX* | *TBB4* |  | *ACT** | *UBP25** | *ACT104** | *UBQ11** | *CYP23** | *PPR** | *FBOX** | *MAPK2** | *r40S* | *TBB4* |
|  |  |  |  | 0.321 | 0.321 | 0.557 | 0.599 | 0.645 | 0.663 | 0.720 | 0.782 | 0.896 | 1.004 |  |  |  | 0.256 | 0.258 | 0.315 | 0.359 | 0.389 | 0.421 | 0.465 | 0.515 | 0.542 | 0.704 |  | 0.928 | 0.904 | 0.877 | 0.853 | 0.821 | 0.760 | 0.637 | 0.576 | 0.493 | 0.242 |
|  |  | 2h-8h |  | *CYP23* | *UBP25* | *PPR* | *ACT104* | *r40S* | *ACT* | *TBB4* | *UBQ11* | *MAPK2* | *FBOX* | 0.17 | 0.16 |  | *MAPK2* | *CYP23* | *ACT104* | *TBB4* | *UBQ11* | *ACT* | *r40S* | *UBP25* | *FBOX* | *PPR* |  | *ACT104** | *UBP25** | *CYP23** | *UBQ11** | *PPR** | *MAPK2* | *r40S*†* | *ACT*†* | *TBB4*†* | *FBOX*†* |
|  |  |  |  | 0.348 | 0.348 | 0.461 | 0.581 | 0.636 | 0.688 | 0.718 | 0.755 | 0.867 | 1.006 |  |  |  | 0.695 | 0.322 | 0.338 | 0.566 | 0.462 | 0.520 | 0.369 | 0.226 | 0.938 | 0.507 |  | 0.986 | 0.939 | 0.900 | 0.803 | 0.784 | 0.078 | 0.947 | 0.935 | 0.933 | 0.587 |
|  |  | 2h-1d |  | *CYP23* | *UBQ11* | *UBP25* | *ACT104* | *TBB4* | *ACT* | *r40S* | *MAPK2* | *PPR* | *FBOX* | 0.11 | 0.18 |  | *UBP25* | *CYP23* | *ACT104* | *UBQ11* | *r40S* | *ACT* | *MAPK2* | *TBB4* | *PPR* | *FBOX* |  | *ACT104** | *UBP25** | *r40S** | *CYP23** | *UBQ11** | *TBB4** | *PPR** | *MAPK2** | *ACT*†* | *FBOX*†* |
|  |  |  |  | 0.240 | 0.240 | 0.314 | 0.532 | 0.617 | 0.660 | 0.701 | 0.746 | 0.780 | 0.976 |  |  |  | 0.198 | 0.305 | 0.338 | 0.374 | 0.420 | 0.444 | 0.475 | 0.476 | 0.507 | 0.944 |  | 0.949 | 0.945 | 0.913 | 0.893 | 0.871 | 0.867 | 0.708 | 0.607 | 0.922 | 0.757 |
|  |  | 8h-1d |  | *ACT104* | *ACT* | *r40S* | *CYP23* | *UBP25* | *UBQ11* | *PPR* | *TBB4* | *MAPK2* | *FBOX* | 0.15 | 0.12 |  | *UBP25* | *CYP23* | *ACT104* | *UBQ11* | *r40S* | *ACT* | *PPR* | *MAPK2* | *TBB4* | *FBOX* |  | *ACT** | *UBP25** | *CYP23** | *r40S** | *ACT104** | *UBQ11** | *TBB4** | *PPR* | *MAPK2* | *FBOX†* |
|  |  |  |  | 0.367 | 0.367 | 0.452 | 0.506 | 0.522 | 0.582 | 0.632 | 0.693 | 0.752 | 0.927 |  |  |  | 0.179 | 0.220 | 0.292 | 0.347 | 0.376 | 0.392 | 0.453 | 0.505 | 0.600 | 0.878 |  | 0.920 | 0.890 | 0.882 | 0.876 | 0.870 | 0.661 | 0.651 | 0.470 | 0.091 | 0.432 |
|  |  | 30min |  | *UBP25* | *FBOX* | *UBQ11* | *PPR* | *CYP23* | *ACT* | *ACT104* | *r40S* | *MAPK2* | *TBB4* | 0.13 | 0.12 |  | *FBOX* | *UBQ11* | *ACT* | *ACT104* | *UBP25* | *MAPK2* | *CYP23* | *PPR* | *r40S* | *TBB4* |  | *UBQ11** | *ACT** | *ACT104* | *UBP25* | *PPR* | *FBOX* | *CYP23* | *MAPK2* | *r40S* | *TBB4†* |
|  |  |  |  | 0.325 | 0.325 | 0.391 | 0.457 | 0.468 | 0.504 | 0.524 | 0.576 | 0.627 | 0.828 |  |  |  | 0.169 | 0.199 | 0.202 | 0.233 | 0.236 | 0.279 | 0.291 | 0.332 | 0.408 | 0.660 |  | 0.970 | 0.849 | 0.803 | 0.745 | 0.744 | 0.654 | 0.568 | 0.334 | 0.237 | 0.399 |
|  |  | 2h |  | *CYP23* | *UBP25* | *UBQ11* | *PPR* | *ACT104* | *TBB4* | *r40S* | *ACT* | *MAPK2* | *FBOX* | 0.09 | 0.09 |  | *UBP25* | *ACT104* | *r40S* | *UBQ11* | *CYP23* | *TBB4* | *MAPK2* | *PPR* | *ACT* | *FBOX* |  | *UBQ11** | *UBP25** | *CYP23** | *PPR* | *MAPK2* | *ACT104*†* | *TBB4*†* | *r40S*†* | *ACT*†* | *FBOX*†* |
|  |  |  |  | 0.206 | 0.206 | 0.262 | 0.317 | 0.545 | 0.659 | 0.720 | 0.774 | 0.830 | 1.012 |  |  |  | 0.245 | 0.289 | 0.413 | 0.414 | 0.420 | 0.457 | 0.509 | 0.527 | 0.566 | 1.027 |  | 0.957 | 0.956 | 0.900 | 0.808 | 0.641 | 0.986 | 0.985 | 0.938 | 0.917 | 0.752 |
|  |  | 8h |  | *ACT* | *r40S* | *ACT104* | *CYP23* | *UBQ11* | *UBP25* | *PPR* | *TBB4* | *MAPK2* | *FBOX* | 0.12 | 0.10 |  | *ACT104* | *UBQ11* | *UBP25* | *CYP23* | *r40S* | *ACT* | *PPR* | *TBB4* | *MAPK2* | *FBOX* |  | *ACT104** | *r40S** | *ACT** | *CYP23** | *UBQ11** | *UBP25** | *PPR** | *FBOX** | *MAPK2** | *TBB4*†* |
|  |  |  |  | 0.262 | 0.262 | 0.338 | 0.384 | 0.433 | 0.460 | 0.505 | 0.591 | 0.727 | 0.850 |  |  |  | 0.163 | 0.193 | 0.207 | 0.227 | 0.310 | 0.396 | 0.485 | 0.661 | 0.707 | 0.880 |  | 0.968 | 0.957 | 0.950 | 0.840 | 0.757 | 0.734 | 0.631 | -0.210 | -0.620 | 0.858 |
|  |  | 1d |  | *CYP23* | *UBQ11* | *MAPK2* | *UBP25* | *PPR* | *ACT104* | *TBB4* | *ACT* | *r40S* | *FBOX* | 0.11 | 0.10 |  | *UBP25* | *CYP23* | *ACT* | *MAPK2* | *UBQ11* | *ACT104* | *r40S* | *PPR* | *TBB4* | *FBOX* |  | *UBP25** | *CYP23** | *ACT** | *MAPK2** | *r40S** | *ACT104** | *UBQ11** | *TBB4** | *PPR** | *FBOX*†* |
|  |  |  |  | 0.215 | 0.215 | 0.298 | 0.365 | 0.469 | 0.529 | 0.554 | 0.594 | 0.633 | 0.861 |  |  |  | 0.149 | 0.193 | 0.285 | 0.313 | 0.356 | 0.356 | 0.433 | 0.447 | 0.465 | 0.839 |  | 0.934 | 0.924 | 0.911 | 0.868 | 0.832 | 0.822 | 0.778 | 0.597 | 0.583 | 0.753 |
