# Supplementary figures and images for: Reference genes selection for Calotropis procera under different salt stress conditions
Source: PLoS One. 2019 Apr 18;14(4):e0215729. doi: 10.1371/journal.pone.0215729 (PMC6472812; doi:10.1371/journal.pone.0215729)

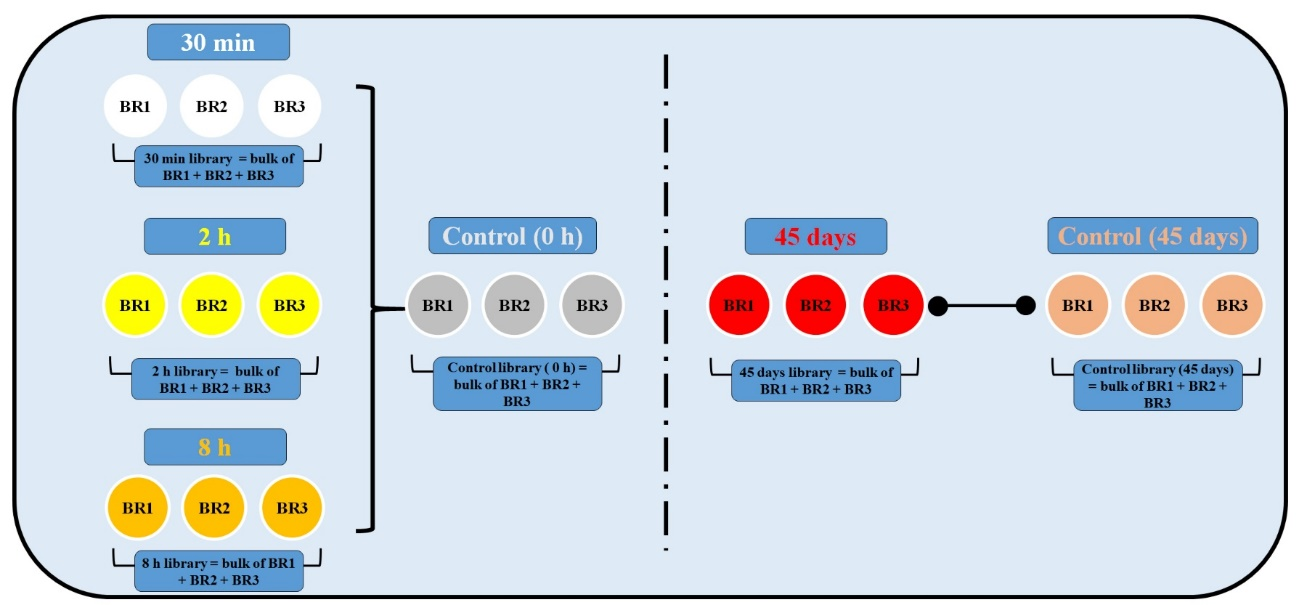

Supplement: S1 Fig — Legend: RB: biological replicate. (TIF) [file pone.0215729.s003.tif]

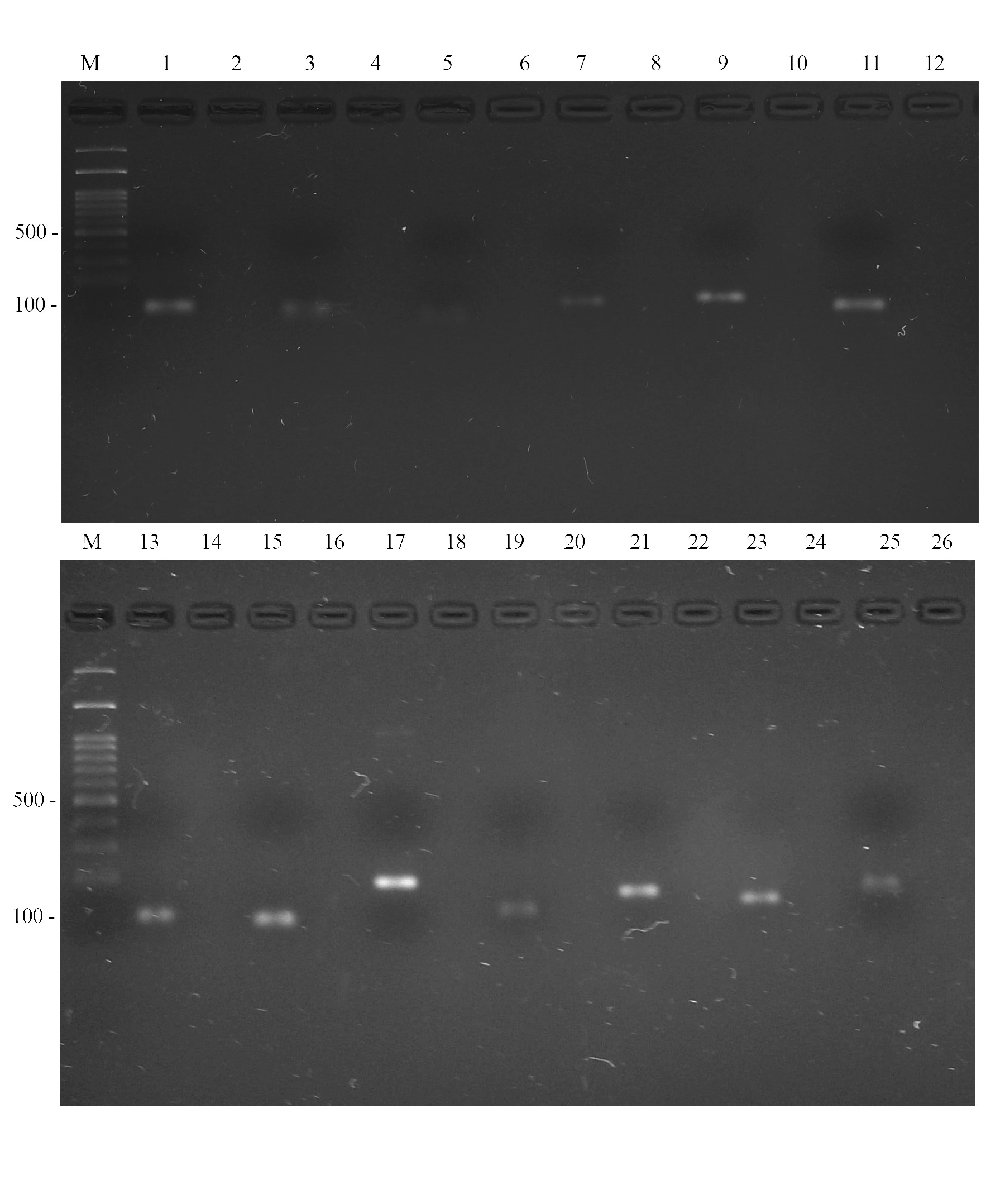

Supplement: S2 Fig — M: marker 100 bp; 1–2 ND1; 3–4 CNBL4; 5–6 NAC78; 7–8 MAPK2; 9–10 CYP23; 11–12 ACT104; 13–14 TBB4; 15–16 UBQ11; 17–18 ACT; 19–20 r40S; 21–22 PPR; 23–24 UBP25; 25–26 FBOX. Even numbers mean no template control. (TIF) [file pone.0215729.s004.tif]

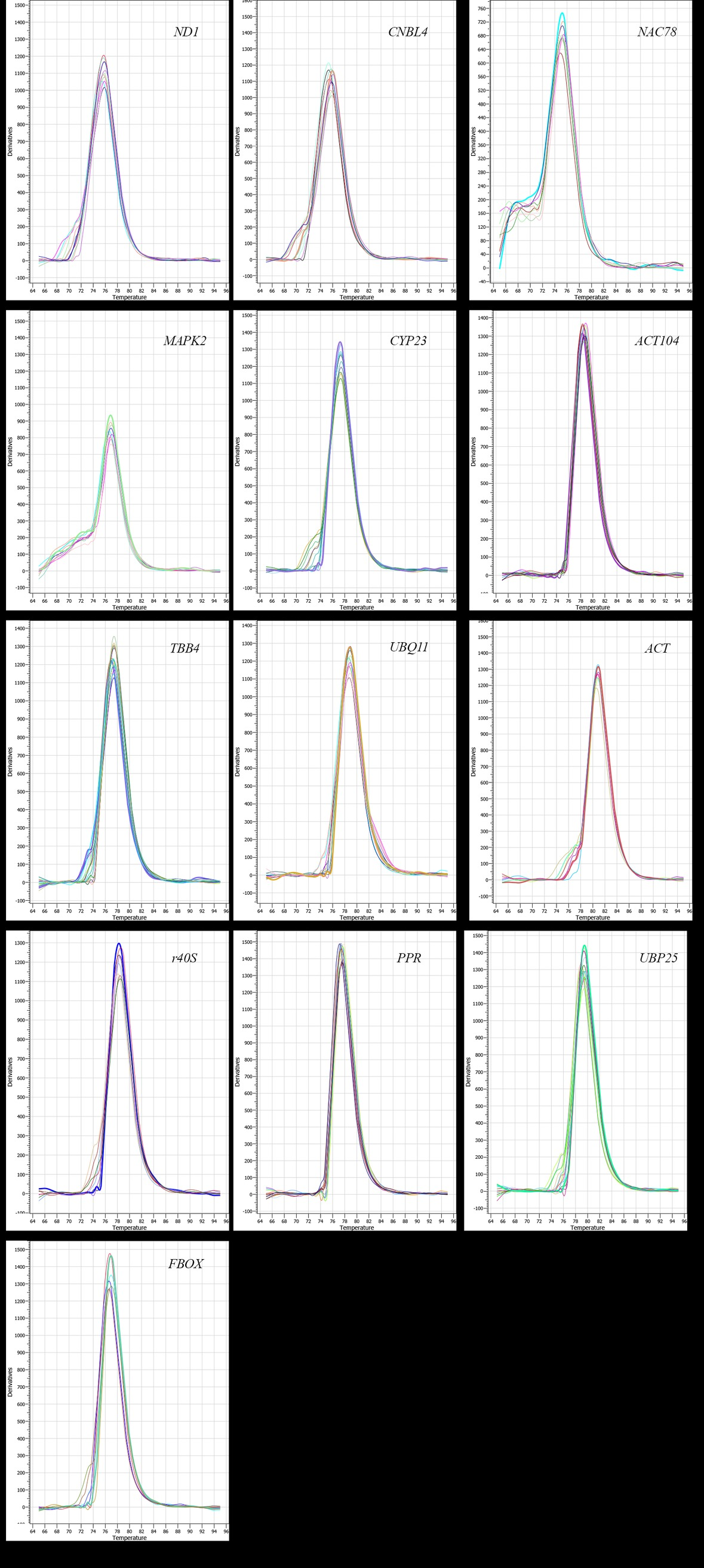

Supplement: S3 Fig — Each line represents the melting curve for each individual replicate. (TIF) [file pone.0215729.s005.tif]
